# The receptor protein tyrosine phosphatase PTPRK promotes intestinal repair and catalysis-independent tumour suppression

**DOI:** 10.1242/jcs.261914

**Published:** 2024-07-22

**Authors:** Katherine A. Young, Katarzyna Wojdyla, Tiffany Lai, Katie E. Mulholland, Silvia Aldaz Casanova, Robin Antrobus, Simon R. Andrews, Laura Biggins, Betania Mahler-Araujo, Philippa R. Barton, Keith R. Anderson, Gareth W. Fearnley, Hayley J. Sharpe

**Affiliations:** ^1^ Signalling programme, Babraham Institute, Cambridge CB22 3AT, UK; ^2^ Cambridge Institute for Medical Research, Hills Road, Cambridge CB2 0XY, UK; ^3^ Bioinformatics, Babraham Institute, Cambridge CB22 3AT, UK; ^4^ Histopathology, Cambridge University Hospitals, Cambridge CB2 0QQ, UK; ^5^ Molecular biology department, Genentech, South San Francisco, CA 94080, USA

**Keywords:** Tyrosine phosphatase, Cell adhesion, Tumour suppression, Epithelial to mesenchymal transition, Signalling

## Abstract

PTPRK is a receptor tyrosine phosphatase that is linked to the regulation of growth factor signalling and tumour suppression. It is stabilized at the plasma membrane by trans homophilic interactions upon cell–cell contact. PTPRK regulates cell–cell adhesion but is also reported to regulate numerous cancer-associated signalling pathways. However, the signalling mechanism of PTPRK remains to be determined. Here, we find that PTPRK regulates cell adhesion signalling, suppresses invasion and promotes collective, directed migration in colorectal cancer cells. *In vivo*, PTPRK supports recovery from inflammation-induced colitis. In addition, we confirm that PTPRK functions as a tumour suppressor in the mouse colon and in colorectal cancer xenografts. PTPRK regulates growth factor and adhesion signalling, and suppresses epithelial to mesenchymal transition (EMT). Contrary to the prevailing notion that PTPRK directly dephosphorylates EGFR, we find that PTPRK regulation of both EGFR and EMT is independent of its catalytic function. This suggests that additional adaptor and scaffold functions are important features of PTPRK signalling.

## Teaser

**Highlighted Article:** PTPRK canonically dephosphorylates tyrosine residues. Deletion in mice supports that it has a function in colon repair and tumour suppression. Use of a catalytic mutant reveals catalysis-independent roles.

## INTRODUCTION

Cell–cell adhesion is essential for normal tissue homeostasis, providing an important barrier function as well as mediating intercellular communication. Through connections to the cytoskeleton, cell–cell adhesion confers mechanical rigidity, yet it must also undergo dynamic remodelling, for example during developmental epithelial to mesenchymal transitions (EMTs) or in response to injury (Bertet et al., 2004; Friedl and Mayor, 2017; Heisenberg and Bellaïche, 2013). Cell–cell adhesion is mediated by transmembrane proteins that span the plasma membrane and link to the underlying cytoskeleton (Garcia et al., 2018). The most well characterized are the classical cadherin complexes, which mediate homophilic interactions and are linked to an intracellular module of αE-, β- and δ-catenins that regulates cadherin stability and provides a connection to the actin cytoskeleton (Nelson, 2008). Dysregulation of cell–cell junctions leads to numerous diseases, including cancer (Cheung and Ewald, 2014; Kaszak et al., 2020). The role of cell–cell adhesion is largely tumour suppressive, which is consistent with contact inhibition of proliferation, a phenomenon whereby cells grown at high density lose responsiveness to growth factors (Fagotto and Gumbiner, 1996; Kim et al., 2011). However, cell junctions must remain intact to facilitate metastasis via collective migration and cell survival in some cancers (Padmanaban et al., 2019). Thus, the complete loss of cell junctions is likely to be deleterious in cancer. In line with this, hybrid EMT states can be more metastatic with worse prognosis than full EMT states (Pastushenko and Blanpain, 2019; Pastushenko et al., 2021). β-catenin (encoded by *CTNNB1*) and p120 catenin (p120^Cat^; a δ-catenin encoded by *CTNND1*) have been linked to cancer growth, EMT and metastasis, and their tyrosine phosphorylation is thought to be oncogenic (Kourtidis et al., 2015; van Veelen et al., 2011). Reversible tyrosine phosphorylation is a major mechanism by which cell junctions can undergo rapid remodelling, mediated by tyrosine kinases and phosphatases (Young et al., 2021).

Several receptor protein tyrosine phosphatases (RPTPs) are implicated as tumour suppressors (Barazeghi et al., 2019; Gu et al., 2019; March et al., 2011; Östman et al., 2006; Starr et al., 2009; Wang et al., 2004) as well as regulators of cell–cell adhesion (Young et al., 2021). The RPTP PTPRK (also known as PTPκ) accumulates at cell–cell contacts, and this accumulation is mediated by pH-dependent formation of a trans homophilic dimer (Hay et al., 2022b). It positively regulates cell–cell junction integrity and directly dephosphorylates δ-catenins, afadin and PARD3 (Fearnley et al., 2019). PTPRK is also a transforming growth factor β1 (TGFβ1) target gene in mammary epithelial cells (Wang et al., 2005), synoviocytes (Stanford et al., 2016) and keratinocytes (Xu et al., 2015), and has been implicated as a tumour suppressor in several cancer types, particularly colorectal cancer (Chen et al., 2015; March et al., 2011; Starr et al., 2009). Computational modelling has previously linked PTPRK with a hybrid EMT state (Selvaggio et al., 2020). In MCF10A cells, depleting PTPRK increases responsiveness to epidermal growth factor (EGF) stimulation but impairs collective cell migration (Wang et al., 2005). In addition to roles in cell–cell adhesion, several oncoprotein substrates have been proposed for PTPRK, including EGFR (Xu et al., 2005), STAT3 (Chen et al., 2015), β-catenin (Anders et al., 2006), CD133 (also known as PROM1; Shimozato et al., 2015) and ZNRF3 (Chang et al., 2020). Thus, PTPRK modulates cell–cell adhesion as well as numerous cancer-associated signalling pathways. The current notion is that PTPRK suppresses cell growth via its phosphatase function.

Forward genetic studies have implicated PTPRK in intestinal tumorigenesis in mice (March et al., 2011; Starr et al., 2009). Truncating variants and a PTPRK-inactivating gene fusion with the Wnt potentiator RSPO3 are also found in human colorectal cancers (Seshagiri et al., 2012). Here, we set out to understand PTPRK function in the mouse colon and in human colorectal cancer cells. We found that PTPRK suppresses invasion, promotes collective migration and supports repair following dextran sulphate sodium (DSS)-induced colitis. Consistent with previous reports, we show that PTPRK is a tumour suppressor in mouse colon; however, using xenografts, we demonstrate that this is surprisingly independent of PTPRK catalytic activity.

## RESULTS

### PTPRK promotes collective migration

To study PTPRK function, we used CRISPR/Cas9 editing to delete it from HT29 and DLD1 human colorectal cancer cells (Fig. S1A). HT29 cells have the highest mRNA levels of wild-type (WT), processed PTPRK (Anders et al., 2006) amongst commonly used cultured colorectal cancer cell lines (Hruz et al., 2008). HT29 cells harbour mutations that activate the Wnt, mitogen-activated protein kinase (MAPK) and phosphoinositide 3-kinase (PI3K) pathways, as well as inactivating mutations in the tumour suppressors *SMAD4* and *TP53* (Ahmed et al., 2013). In sub-confluent cell culture, PTPRK knockout (KO) HT29 cells appeared more scattered and more frequently displayed a spindle-like morphology with multiple projections compared to WT HT29 cells (Fig. S1B,C). In confluent co-cultures of WT and PTPRK KO HT29 cells, the fluorescence intensity of p120^Cat^ staining was lower at junctions between KO cells than at junctions between WT cells (Fig. S1D,E). This indicates altered p120^Cat^ levels at junctions, which might reflect altered p120^Cat^ phosphorylation in PTPRK KO cells (Kourtidis et al., 2013) or impaired cell junctions. As cell density increases, p120^Cat^ tyrosine phosphorylation at Y904 decreases in numerous cell lines (Lampugnani et al., 1997) (Fig. S1F); this is likely to reflect the accumulation of density-regulated RPTPs, including PTPRK (Fearnley et al., 2019), that dephosphorylate this site (Young et al., 2021). In unstimulated conditions, p120^Cat^ was hyperphosphorylated in PTPRK KO HT29 cells, with a greater effect observed at cell higher density (Fig. S1F,G). Thus, deleting PTPRK from colorectal cancer cells leads to altered adhesive signalling and morphological changes resembling a more mesenchymal state, similar to previous findings in MCF10A cells (Fearnley et al., 2019).

**Fig. 1.**
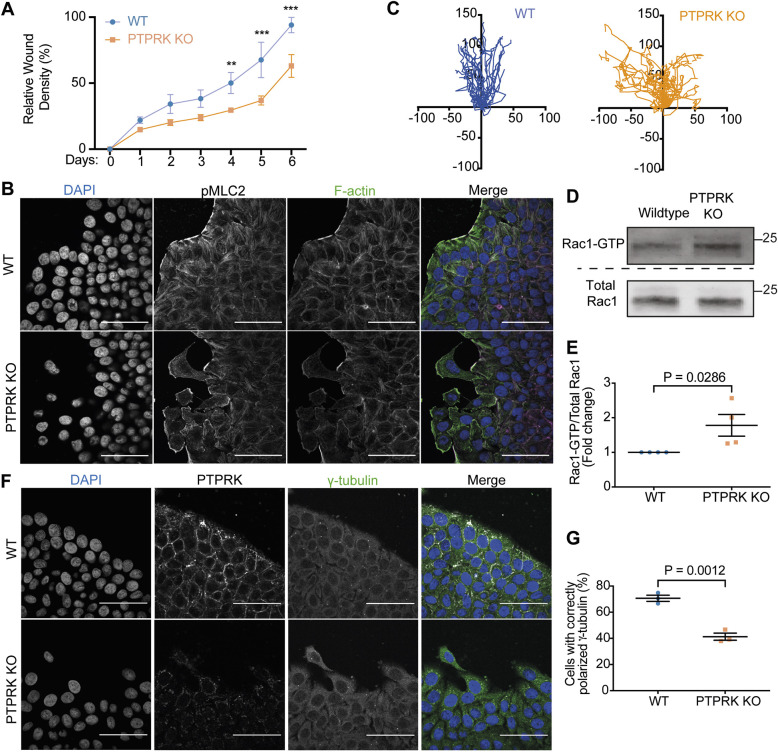
**PTPRK supports collective migration and suppresses Rac.** (A) Quantification of relative ratio of the occupied area to the total area of the initial scratched region (wound density) over 6-day scratch wound experiments performed using WT and PTPRK KO HT29 cells. Scratch wound closure was tracked using the Incucyte Scratch Wound Analysis software module (*n*=3). Mean±s.d. Unpaired, two-tailed *t*-test: **P≤0.01, ***P≤0.001. (B) Immunofluorescence microscopy analysis of the scratch wound leading edge of WT and PTPRK KO HT29 cells stained for myosin regulatory light chain 2 phosphorylated at S20 (pMLC2), F-actin (phalloidin; green in merge image) and DNA (DAPI; blue in merge image). Confocal microscopy images shown are representative of three experiments. Scale bars: 50 µm. (C) Movement of HT29 cells on the leading edge was tracked using ImageJ analysis of Incucyte images taken every 2 h over 48 h (*N*=10 cells per experiment; *n*=3). Distances are shown in μm. (D) GTP-bound Rac1 was pulled down using GST fusion proteins, corresponding to the p21-binding domain (PBD) of human PAK1. Inputs (1% total) and pulldowns were subjected to immunoblot analysis with an anti-Rac1 antibody. Molecular masses are shown in kDa. Representative blots are shown. (E) Quantification of the ratio of GTP-Rac1 to total Rac1 on immunoblots as described in D (*n*=4). Mean±s.d. Unpaired, two-tailed *t*-test. (F) Representative images of leading-edge cells stained for PTPRK and the centrosome marker γ-tubulin (green in merge image), with DNA stained using DAPI (blue in merge image). Scale bars: 50 µm. (G) Quantification of γ-tubulin polarization (see Materials and Methods for definition of correct polarization). *N*=3 fields of view per experiment; *n*=3. Mean±s.d. Unpaired, two-tailed *t*-test.

PTPRK has previously been shown to be required for TGFβ-induced wound closure of HER2 (ERBB2)-overexpressing MCF10A cells (Wang et al., 2005). Using a scratch wound assay under unstimulated growth conditions where WT and PTPRK KO HT29 cells showed similar proliferation rates (Fig. S1H), the PTPRK KO HT29 cells displayed impaired ‘wound’ healing compared to that of WT HT29 cells (Fig. 1A; Fig. S1I). We noted that PTPRK KO HT29 cells at the leading edge more readily detached from the main cell mass (Fig. 1B) and, when tracked over a 48 h time period, showed a random migration pattern (Fig. 1C; see Movies 1 and 2 for WT and PTPRK KO, respectively). This is comparable to the effects of knockdown of cell junction proteins, including p120^Cat^ (Hernández-Martínez et al., 2019). Active, GTP-bound Rac1 promotes random migration over persistent directed migration (Pankov et al., 2005) but is also required for coordinated collective migration (Zegers and Friedl, 2014). Furthermore, p120^Cat^ regulates the balance between activities of Rho and Rac GTPases at lateral junctions (Wildenberg et al., 2006). We therefore tested whether PTPRK regulates Rac1 signalling. Levels of GTP-bound Rac1 were increased in unscratched PTPRK KO HT29 cells compared to the levels in WT HT29 cells (Fig. 1D,E), suggesting that PTPRK suppresses Rac1 at cell contacts, which in turn suppresses cell protrusions (Capuana et al., 2020). Directed migration also requires correct front–rear polarity (Kupfer et al., 1982; Luxton and Gundersen, 2011; Ueda et al., 1997). For example, the PTPRK substrate PARD3 is required for collective migration (Pegtel et al., 2007; Pinheiro and Montell, 2004; Schmoranzer et al., 2009) and invasion (Hidalgo-Carcedo et al., 2011). Consistent with a polarity defect, PTPRK KO HT29 cells at the leading edge exhibited reduced anterior γ-tubulin localization, indicating altered polarization compared to that of WT HT29 cells (Fig. 1F,G). Thus, PTPRK is required for directed, coordinated migration and regulates Rho family GTPases and correct polarization. Since epithelial barrier integrity and wound healing support responses to damage, and because PTPRK has been linked genetically to coeliac disease (Trynka et al., 2011), we next sought to determine whether PTPRK is required for injury recovery in the colon.

### PTPRK supports recovery from DSS-induced colitis

**Fig. 2.**
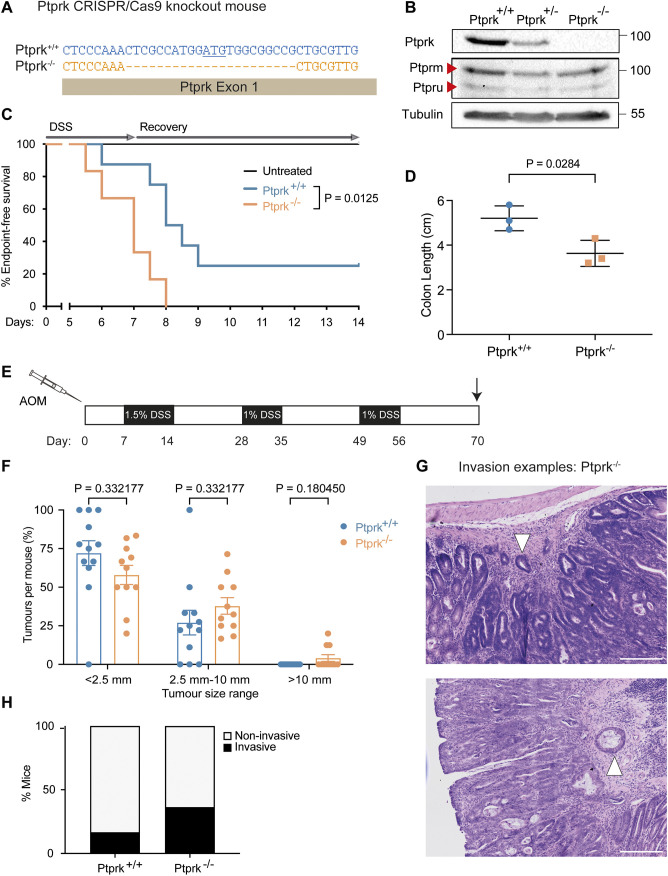
**PTPRK supports recovery from damage and suppresses tumour growth and invasion *in vivo*.** (A) Schematic of the CRISPR-targeted 22 bp deletion in *Ptprk* exon 1 to disrupt expression of PTPRK. The start ATG codon is underlined. (B) Immunoblot analysis of colon tissue lysates from *Ptprk^+/+^*, *Ptprk^+/−^* and *Ptprk^−/−^* mice using antibodies recognizing PTPRK in addition to related members of the R2B family of RPTPs: PTPRM (also known as PTPµ) and PTPRU (also known as PTPϕ). Molecular masses are shown in kDa. Blots shown are representative of three mice. (C) DSS treatment regimen and Kaplan–Meier curves for untreated *Ptprk^+/+^* mice (*n*=5), and DSS-treated *Ptprk^+/+^* (*n*=8) and *Ptprk*^−/−^ (*n*=8) mice over the 14-day experiment. Endpoints as described in the Materials and Methods. Log-rank (Mantel–Cox) test. (D) Colon lengths of mice measured on day 7 following DSS-induced colitis. *Ptprk^+/+^, n*=3; *Ptprk*^−/−^, *n*=3. Mean±s.d. Unpaired, two-tailed *t*-test. (E) AOM–DSS colorectal cancer model treatment regimen. (F) Individual tumour sizes from each *Ptprk^+/+^* (*n*=12) and *Ptprk*^−/−^ (*n*=11) mouse colon. Mean±s.e.m. Unpaired, two-tailed *t*-test. (G) Representative images of *Ptprk*^−/−^ mouse colorectal tumour that has invaded into submucosa (indicated by white arrowheads). Haematoxylin and eosin staning. Scale bars: 200 μm. (H) Graph of percentage of mouse colons displaying tumour invasion into submucosa. *Ptprk^+/+^*, *n*=12; *Ptprk*^−/−^, *n*=11. Analyses in F–H were conducted by a pathologist who was unaware of the treatment groups.

To investigate the *in vivo* function of PTPRK, we generated a 22 bp deletion in *Ptprk* exon 1 using CRISPR/Cas9 editing in the mouse germline (Fig. 2A). *Ptprk^−/−^* mice no longer express PTPRK protein, as determined by immunoblotting of colon lysates (Fig. 2B). There were no overt phenotypes in adult *Ptprk^−/−^* mice, including fertility since litters produced expected mendelian ratios of genotypes (Fig. S2A,B). Although the difference was not statistically significant, both 6-week-old males and females trended towards having a lower body mass (Fig. S2C,D). We aged a cohort of mice to 11 months and subjected them to an assessment by an independent pathologist (Table S1). A homozygous male showed a possible cataract, similar to a phenotype since reported by the international mouse phenotyping consortium (https://www.mousephenotype.org/data/genes/MGI:103310). The only other additional finding from the consortium was a hyperactivity phenotype. Focusing on the colon, there were no clear differences in the overall length (Fig. S2E,F) or cellular architecture (Fig. S2G,H) of *Ptprk^−/−^* mouse colons compared to those of WT littermate controls. Given that there are three additional paralogues of PTPRK in mice, compensation or redundancy during development is a possibility. Indeed, the paralogous proteins PTPRM and PTPRU were detectable in *Ptprk^−/−^* colons (Fig. 2B).

We reasoned that challenging the *Ptprk^−/−^* mice could reveal a non-redundant function of PTPRK. *PTPRK* has previously been found to be downregulated in coeliac disease colons (Bondar et al., 2014; Nanayakkara et al., 2022), indicating a potential function in colon injury responses (Ihara et al., 2017). Therefore, to test the *in vivo* role of PTPRK, we used the well-established DSS colitis model, which tests the integrity of intestinal immune function (Kiesler et al., 2015) and epithelial barrier (De Arcangelis et al., 2017; Fan et al., 2019; Tanaka-Okamoto et al., 2011). To determine whether PTPRK could regulate the immune response to the DSS colitis challenge, we explored its function in T lymphocytes. PTPRK has previously been linked to T cell development based on a spontaneous deletion in rats (Erdenebayar et al., 2009; Iwata et al., 2010). However, in this rat strain, *Ptprk* is co-deleted with *Themis*, which regulates T cell development (Yang et al., 2022) and could explain the phenotypes observed (Stanford et al., 2012). Proteomic quantification revealed that PTPRK is not highly expressed in mouse immune cells, other than activated CD8-positive T cells (Fig. S2I) (Brenes et al., 2023). We found no significant differences in numbers of mature T cells isolated from the spleens of WT and *Ptprk*^−/−^ littermates (Fig. S2J). Furthermore, CD8-positive cytotoxic T cell killing was similar between cells isolated from WT and *Ptprk*^−/−^ littermates (Fig. S2K), suggesting that PTPRK is not a critical determinant of T cell development or cytotoxic effector function. We therefore make the assumption that the DSS colitis model predominantly assesses the impact of PTPRK loss on epithelial barrier integrity and/or repair capacity.

Since female mice are largely refractory to DSS-induced colitis (Bábíčková et al., 2015), we treated cohorts of male mice with 1.5% DSS in drinking water for 7 days and tracked their recovery for an additional 7 days without DSS. *Ptprk* deletion resulted in a greater susceptibility to DSS-induced damage, which was measured by the time taken for mice to reach set humane endpoints, including greater than 15% weight loss (Fig. 2C). We also compared colon lengths, an indicator of colon damage, after 7 days of treatment. At this time point, *Ptprk^−/−^* colons were significantly shorter than *Ptprk^+/+^* colons, indicating increased susceptibility to damage or decreased repair (Fig. 2D). Taken together, our data support a role for PTPRK in wound healing and injury repair in cancer cells and in mice.

### PTPRK suppresses tumour growth *in vivo*

To test PTPRK tumour suppressor function directly in mice, we implemented the azoxymethane (AOM)–DSS colorectal cancer model (Neufert et al., 2007). Following a single intraperitoneal injection of the carcinogen (AOM), male mice were subjected to three cycles of DSS treatment (Fig. 2E). The combination of the mutagen and intestinal inflammation triggered the growth of colorectal tumours. Following a histopathological analysis of colons conducted by a researcher unaware of the treatment groups, we found that *Ptprk*^−/−^ mice exhibited a trend towards larger and more invasive tumours in comparison to those of WT littermate controls (Fig. 2F–H). Taken together, these data indicate that PTPRK suppresses tumour growth and invasion *in vivo*.

### Suppression of tumour growth and invasion by PTPRK is independent of catalytic activity

**Fig. 3.**
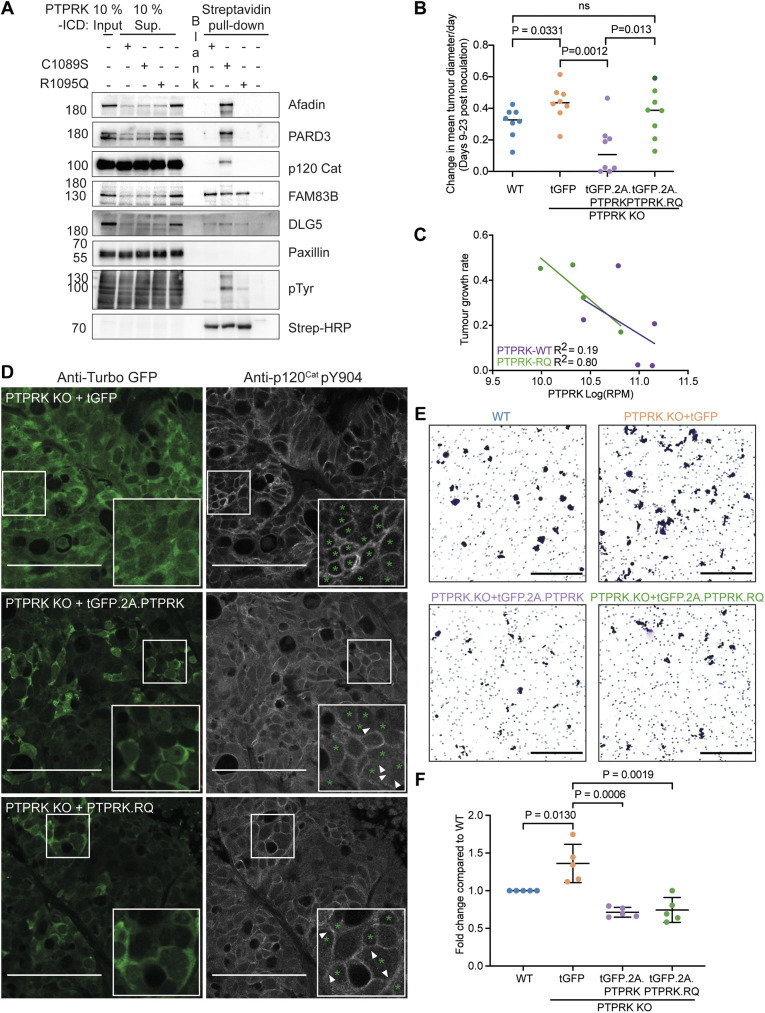
**PTPRK suppresses HT29 tumour growth and invasion.** (A) The indicated recombinant PTPRK ICDs bound to streptavidin resin were used in pull-down assays from pervanadate treated MCF10A cell lysates. After extensive washing, bound proteins were eluted in sample buffer and analysed by immunoblotting with the indicated antibodies. Molecular masses are shown in kDa. pTyr, phosphotyrosine; Strep-HRP, HRP-conjugated streptavidin; Sup., supernatant. Blots shown are representative of three or more experiments. (B) Tumour growth rate (change in mean diameter/day) of the indicated HT29 xenografts over 14 days (days 9–23 with the exception of one PTPRK.RQ-expressing tumour that reached the endpoint size on day 21, indicated by the dark green datapoint). WT, *n*=8; PTPRK KO with expression of tGFP, *n*=8; PTPRK KO with expression of tGFP and PTPRK, *n*=6; PTPRK KO with expression of tGFP and PTPRK.RQ, *n*=7. Horizontal lines indicate the mean. Unpaired, two-tailed *t*-test (ns, not significant). (C) Correlation of tumour growth rate (as described in B) and PTPRK transcript levels for PTPRK KO xenografts expressing either WT PTPRK (purple; *n*=4) or PTPRK.RQ (green; *n*=5) using simple linear regression. Goodness of fit is shown as R^2^. RPM, reads per million mapped reads. (D) Immunofluorescence analysis of tumour xenografts stained with antibodies to detect tGFP and phosphorylation of p120^Cat^-Y904, as well as DAPI (latter not shown). Top, PTPRK KO with expression of tGFP only; middle, PTPRK KO with expression of WT PTPRK; bottom, PTPRK KO with expression of PTPRK.RQ. Boxes mark regions shown in insets. Doxycycline-induced tGFP marks cells with higher levels of expression of PTPRK (green asterisks). White arrowheads indicate suppression of p120^Cat^-Y904 phosphorylation between adjacent PTPRK-expressing cells. Scale bars: 100 µm. Images are representative of three fields of view for three tumours. (E) Representative images of Crystal Violet-stained Transwells showing invasion of the indicated HT29 cell lines through Matrigel-coated inserts. Scale bars: 500 µm. (F) Quantification of invasion of the indicated HT29 cell lines through Matrigel-coated Transwell inserts after 48 h. Mean±s.d. (*n*=5). Unpaired, two-tailed *t*-test.

We and others have identified numerous PTPRK substrates that have been implicated in PTPRK tumour suppressor function (Chang et al., 2020; Fearnley et al., 2019; Matsushita et al., 2019). To investigate the role of PTPRK phosphatase activity in tumour suppression, we used a rescue approach in PTPRK KO HT29 xenografts. Mutating key residues in the core catalytic HCSxxxxR motif disrupts protein tyrosine phosphatase (PTP) catalytic activity (Kolmodin and Åqvist, 2001). Mutating the PTPRK catalytic cysteine (C1089S) has substrate-trapping properties that could have dominant-negative effects (Fearnley et al., 2019). In contrast, we found that mutating the catalytic arginine (R1095) also abolishes recombinant protein catalytic activity in an *in vitro* phosphatase assay (Fig. S3A,B). To investigate substrate trapping by the PTPRK catalytic mutants, we performed pull-down assays using pervanadate-treated MCF10A cell lysates and recombinant PTPRK intracellular domains (ICDs). We found that the C1089S mutant PTPRK ICD displayed increased binding to known PTPRK substrates – including afadin, p120^Cat^ and PARD3 – compared to the WT ICD. However, the R1095Q mutant PTPRK ICD showed similar binding to that of the WT ICD and did not appear to be a substrate trap. However, it retained the ability to bind interactors on its non-catalytic D2 domain, including FAM83B and DLG5 (Fig. 3A). The negative control, paxillin, did not bind to any PTPRK proteins. Therefore, to investigate the effects of catalysis alone, we opted to use the R1095Q mutant. We next generated stable PTPRK KO HT29 cell lines for doxycycline-inducible expression of PTPRK or the R1095Q catalytic mutant (hereafter referred to as PTPRK.RQ). We confirmed that the constructs were doxycycline inducible and co-expressed TurboGFP (tGFP; Fig. S3C). As previously observed, reintroduction of PTPRK into PTPRK KO cells suppressed p120^Cat^-Y904 phosphorylation. In contrast, the PTPRK.RQ mutant was less effective at suppressing p120^Cat^-Y904 phosphorylation than WT PTPRK (Fig. S3C,D). Thus, we have established cell lines to assess the impact of PTPRK and its phosphatase activity on colorectal cancer cell behaviours.

To evaluate the role of PTPRK phosphatase activity on tumour growth we generated xenografts. HT29 cell lines were sorted for equivalent tGFP expression levels following doxycycline induction and injected subcutaneously into the flanks of nude mice that were maintained with doxycycline-containing drinking water. Consistent with a tumour suppressor function, we found that PTPRK KO xenografts had a higher growth rate than WT xenografts, and that this could be fully rescued by re-expressing PTPRK (Fig. 3B). The phosphatase activity-deficient mutant displayed an intermediate phenotype, suppressing growth to similar levels to the original WT line but to a lesser extent than reintroduced WT PTPRK (Fig. 3B). To investigate PTPRK signalling further, we subjected tumours to RNA sequencing (RNA-seq), histology and phosphotyrosine proteomic analyses.

The RNA-seq analysis enabled us to confirm the presence of the R1095Q mutation in relevant samples (Fig. S3E), and that PTPRK.RQ was expressed at lower levels than PTPRK (Fig. 3C; Fig. S3F). Tumour growth rate inversely correlated with both PTPRK and PTPRK.RQ expression levels (Fig. 3C), suggesting that PTPRK tumour suppression is at least in part independent of phosphatase activity. We next evaluated the ability of PTPRK and PTPRK.RQ to suppress p120^Cat^-Y904 phosphorylation in the xenografts, which is present at cell junctions. Using GFP as a proxy for PTPRK expression (Fig. 3D, green asterisks) we observed suppression of p120^Cat^-Y904 phosphorylation between adjacent PTPRK-expressing cells (Fig. 3D, white arrowheads; Fig. S3G). In contrast, there was very little suppression of p120^Cat^-Y904 phosphorylation by PTPRK.RQ. This was evident from fluorescence intensity line scans across junctions, where the PTPRK KO and PTPRK.RQ tumours show clear peaks of p120^Cat^-Y904 phosphorylation, whereas the PTPRK cells show no peak in fluorescence intensity (Fig. S3G). These images also revealed heterogeneity in expression of tGFP in PTPRK- and PTPRK.RQ-expressing tumours, compared to the PTPRK KO tumours, despite prior flow cytometry enrichment of tGFP-expressing cells. This could indicate selective pressure against PTPRK and PTPRK.RQ expression, yielding the diversity of growth rates in ‘rescued’ cell line xenografts (Fig. 3B).

We next explored the role of PTPRK phosphatase activity in invasion, given the increased instances of invasive tumours in AOM–DSS-treated *Ptprk*^−/−^ mice. First, we cultured PTPRK KO and WT HT29 cells as tumour spheres (Fig. S3H) (Weiswald et al., 2015). PTPRK KO cells displayed more projections into the surrounding agarose than WT cells, as quantified by measuring sphere circularity (Fig. S3I). The PTPRK KO HT29 cells invaded into Matrigel more readily than the WT HT29 cells. This was completely rescued by reintroduction of PTPRK or PTPRK.RQ (Fig. 3E,F). Thus, both tumour growth and invasion suppression by PTPRK appear to be independent of catalytic activity.

### PTPRK regulation of gene transcription

**Fig. 4.**
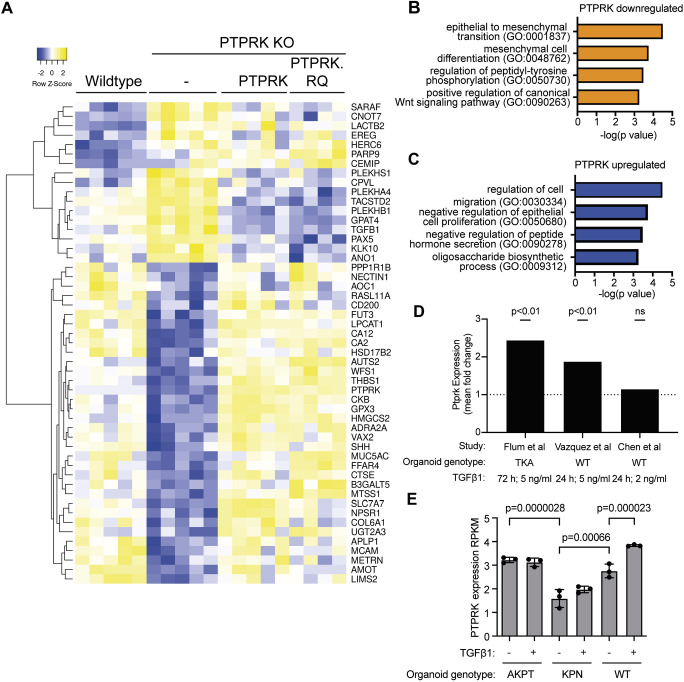
**PTPRK tumour suppression is only partially dependent on catalytic activity.** (A) Heatmap showing differential gene expression between WT and PTPRK KO HT29 cells that was rescued by reintroduction of PTPRK and/or PTPRK.RQ. (B) GO analysis of biological processes of genes downregulated by PTPRK, performed using Enrichr. (C) GO analysis of biological processes of genes upregulated by PTPRK, performed using Enrichr. (D) Fold change *Ptprk* expression upon treatment with indicated times and amounts of TGFβ1, based on RNA-seq analysis from the indicated studies. Unpaired, two-tailed *t*-test (ns, not significant). TKA, organoids derived from *Apc*^580S/580S^ *Kra*s^LSL-G12D/+^ *Trp53*^LSL-R172H/+^ TgVillin^CreERT2^ mice. (E) *Ptprk* expression based on featureCounts output from Gil Vazquez et al. (2022). AKPT, *villin*^CreER^ *Apc*^fl/fl^ *Kras*^G12D/+^ *Trp53*^fl/fl^ *Tgfbr1*^fl/fl^. KPN, *villin*^CreER^ *Kras*^G12D/+^ *Trp53*^fl/fl^ *Rosa26*^N1icd/+^. Mean±s.d. *n*=3. Wald test in DESeq2. RPKM, reads per kilobase per million mapped reads.

To gain insight into the impact of modulating PTPRK in xenografts, we performed differential gene expression analysis on our bulk RNA-seq data (Fig. S4A). Comparing WT and PTPRK KO samples, there were 44 differentially expressed genes that were subsequently rescued by reintroduction of PTPRK and/or PTPRK.RQ (Fig. 4A; Table S2). Amongst these genes, there were no genes that were statistically significantly differentially regulated between PTPRK and PTPRK.RQ samples (Fig. S4B,C). Thus, it appears that PTPRK-regulated gene expression changes were driven largely by phosphatase-independent signalling. Gene ontology (GO) analysis of genes suppressed by PTPRK (performed using Enrichr; Xie et al., 2021) revealed overrepresentation of genes annotated with the biological processes ‘epithelial to mesenchymal transition’ and ‘mesenchymal cell differentiation’, as well as Wnt signalling (Fig. 4B). Since HT29 cells lack SMAD4 function, this indicates that PTPRK is a putative negative regulator of SMAD4-independent TGFβ and EMT signalling. Amongst genes that were upregulated by PTPRK, GO analysis revealed overrepresentation of genes annotated with the terms ‘negative regulation of epithelial cell proliferation’ and ‘regulation of cell migration’ (Fig. 4C). This is consistent with both PTPRK and PTPRK.RQ suppressing tumour growth and invasion. Several of the gene changes observed in xenografts could be reproduced by reverse transcription–quantitative PCR (RT-qPCR) in two-dimensional (2D) culture (Fig. S5A,B). Amongst these genes, none were statistically significantly differentially regulated between PTPRK and PTPRK.RQ samples. However, when we carried out a pairwise comparison between PTPRK KO xenografts re-expressing PTPRK or PTPRK.RQ there were 19 differentially regulated genes (Table S2). This included *AFDN*, which encodes the PTPRK substrate afadin (Hay et al., 2022a), and genes linked to oxidation and metabolism, including *FOXO3*, *AQP3* and *UGDH*. This indicates that PTPRK phosphatase-dependent signalling might affect metabolic processes. Overall, our data reveal that PTPRK suppresses growth and EMT independently of catalysis.

Since PTPRK suppresses *TGFB1* expression (Fig. 4A)*,* we were curious whether PTPRK is part of a feedback loop, having been identified as a target of SMAD4 in MCF10A cells previously (Wang et al., 2005). Colorectal cancer cell lines typically harbour inactivating mutations in TGFβ pathway components, precluding such an analysis. However, three recent publications have performed RNA-seq on intestinal organoids treated with and without TGFβ1 (Chen et al., 2023; Flum et al., 2022; Gil Vazquez et al., 2022). A modest, but statistically significant, ∼2-fold upregulation of *Ptprk* was observed for organoids treated with TGFβ1 in the RNA-seq data from two of these studies (Fig. 4D). After reanalysing RNA-seq data for multiple genotypes (Gil Vazquez et al., 2022), we observed that organoids mutant for APC, P53 (also known as TP53 or TRP53 in mice), KRas and TGFβ receptor show similar levels of *Ptprk* to wildtype. However, organoids mutant for P53 and KRas harbouring the Notch1 ICD showed significantly reduced levels of PTPRK (Fig. 4E). These analyses reveal that in intestinal organoids *Ptprk* expression is modestly upregulated by TGFβ1 and potentially downregulated by Notch signalling.

### PTPRK restricts EGFR signalling independently of catalytic activity

**Fig. 5.**
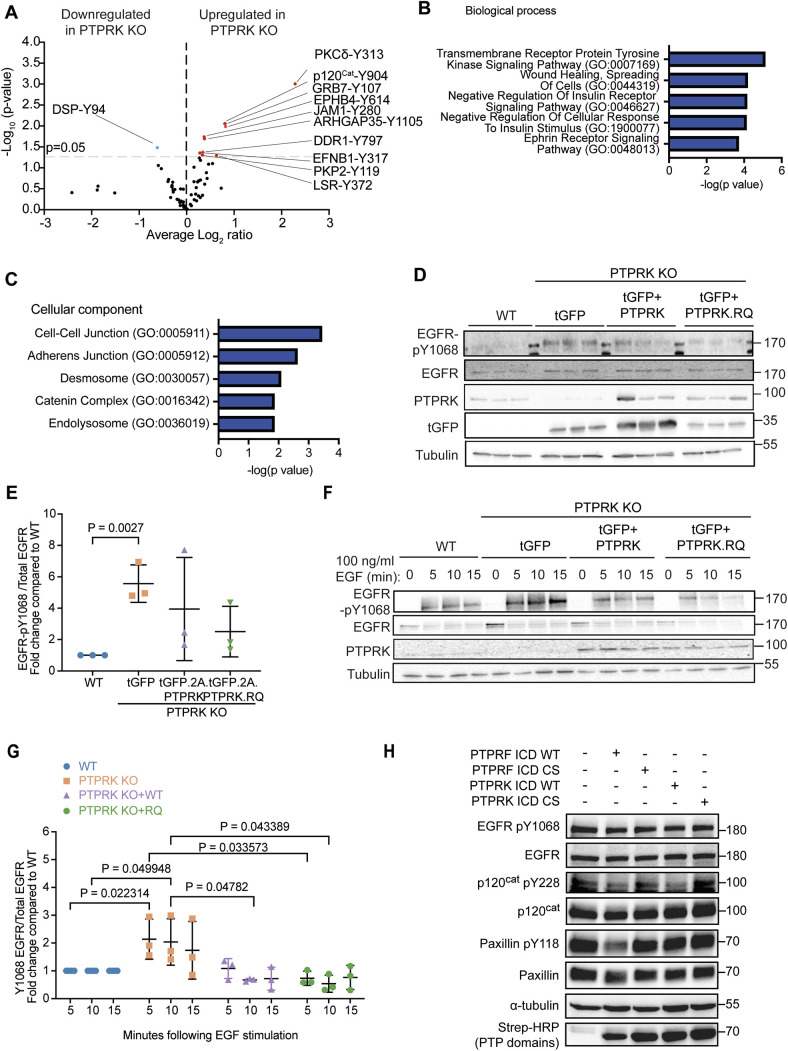
**PTPRK restrains tyrosine phosphorylation of cell adhesion and RTK signalling pathway components.** (A) Volcano plot of tyrosine phosphosites detected in PTPRK KO xenografts compared to WT xenografts. Phosphosites more than 50% enriched in PTPRK KO samples are marked in red and those more than 50% enriched in WT are marked in blue (*P*<0.05 significance threshold for enrichment, grey dashed line; *n*=3). Significance analysis was carried out in Perseus using *t*-test and permutation-based FDR to correct for multiple hypothesis testing. FDR=0.01. (B) GO term analysis of proteins with significantly increased tyrosine phosphorylation in PTPRK KO xenografts (as defined in A) using PANTHER biological process annotation set. *P*<0.05. (C) GO term analysis of proteins with significantly increased tyrosine phosphorylation in PTPRK KO xenografts (as defined in A) using PANTHER cellular component annotation set. *P*<0.05. (D) Lysates from the indicated xenograft homogenates were subjected to immunoblot analysis with indicated antibodies. EGFR-pY1068, EGFR phosphorylation at Y1068. Each lane represents an independent xenograft. Blots shown are representative of three experiments. (E) Densitometric quantification of EGFR Y1068 phosphorylation normalized against total EGFR in the indicated xenografts prior to treatment. Mean±s.e.m. (*n*=3). Unpaired, two-tailed *t*-test: *P* value shown for comparison with *P*≤0.05. All other comparisons *P*>0.05. (F) WT HT29 cells or PTPRK KO HT29 cells with stably integrated doxycycline-inducible tGFP, PTPRK or PTPRK.RQ, as indicated, were cultured for 4 days in 1 µg/ml doxycycline, starved overnight and treated with EGF (100 ng/ml) for the stated time points, followed by lysis and immunoblot analysis with indicated antibodies. (G) Densitometric quantification of EGFR Y1068 phosphorylation normalized against total EGFR, as assayed in F. Mean±s.e.m. (*n*=3). Multiple unpaired *t*-tests: *P* values shown for comparisons with *P*≤0.05. All other comparisons *P*>0.05. (H) Pervanadate-treated MCF10A lysates were incubated with and without recombinant ICDs from PTPRF and PTPRK for 30 min at 4°C prior to immunoblot analysis with the indicated antibodies. ICDs were either WT or had catalytic cysteine mutations (CS; C1548S for PTPRF, C1089S for PTPRK), as indicated. Blots shown are representative of three experiments. In D, F and H, molecular masses are shown in kDa.

To determine additional mechanisms of tumour suppression by PTPRK, we obtained quantitative tyrosine phosphoproteomes of WT and PTPRK KO xenograft tumour samples (Fig. 5A). We quantified 73 phosphosites, of which 11 were significantly differentially regulated in PTPRK KO and WT tumours. The most enriched biological processes amongst the ten upregulated phosphosites in PTPRK KO xenografts were related to cell adhesion and receptor tyrosine kinase (RTK) signalling (Fig. 5B,C). Several of the phosphoproteins identified [including sites on EphB4, p120^Cat^, ARHGAP5 (a paralogue of ARHGAP35, both p190RhoGAPs) and PKP2] have previously been found to be modulated by PTPRK in MCF10A cells (Fearnley et al., 2019). PKP2 and p120^Cat^ have also been identified as interactors and direct PTPRK substrates. However, we found that ARHGAP35 is not a PTPRK ICD interactor (Fig. S6A). ARHGAP35 phosphorylation by Src on Y1105 is linked to activation of its Rho GTPase activating function and is regulated by p120^Cat^ (Wildenberg et al., 2006). Using publicly available data (de Bruijn et al., 2023), we found that levels of p120^Cat^-Y904 phosphorylation correlate with levels of ARHGAP35-Y1105 phosphorylation in human colorectal cancer samples (Fig. S6B), indicating that the previously reported co-dependence of these proteins could be mediated by phosphorylation (Wildenberg et al., 2006). The remaining upregulated phosphosites were associated with RTKs or proteins that function downstream of RTKs, including PKCδ-Y313 (Singh et al., 2009) and GRB7-Y107 (Chu et al., 2019).

PTPRK, like many RPTPs, has been linked to EGFR signalling (Tarcic et al., 2009). Phosphorylation of EGFR-Y1197, an EGFR autophosphorylation site, was on average 1.15-fold higher in the PTPRK KO xenografts than in WT xenografts (Table S3). By immunoblotting, we identified increased phosphorylation of EGFR-Y1068 in PTPRK KO xenografts compared to levels in WT xenografts, and this could be suppressed by both PTPRK and PTPRK.RQ re-expression (Fig. 5D,E). Overexpression of PTPRK did not fully repress phosphorylation of EGFR-Y1068; however, this could reflect the mosaicism in expression observed by immunofluorescence (Fig. 3D). In 2D culture, PTPRK KO HT29 cells showed increased sensitivity to EGF stimulation (Fig. 5F,G) but no clear changes in baseline EGFR-Y1068 phosphorylation. DLD1 and MCF10A PTPRK KO cells also exhibited enhanced responses to EGF compared to the responses of WT cells (Fig. S6C–F). As observed for the xenografts, induced expression of PTPRK and PTPRK.RQ in PTPRK KO HT29 cells dampened the responsiveness to EGF (Fig. 5F,G). This was an unexpected result since PTPRK has been proposed to directly dephosphorylate EGFR (Xu et al., 2005). To test whether the PTPRK PTP domains can mediate direct dephosphorylation of phosphorylated EGFR-Y1068, we used an in-lysate dephosphorylation assay. Briefly, quenched, pervanadate-treated MCF10A cell lysates served as a pool of substrates for recombinant PTPRK and PTPRF PTP domains. Under conditions where PTPRK dephosphorylates its known substrate p120^Cat^, EGFR was not dephosphorylated by any of the RPTPs assayed (Fig. 5H).

**Fig. 6.**
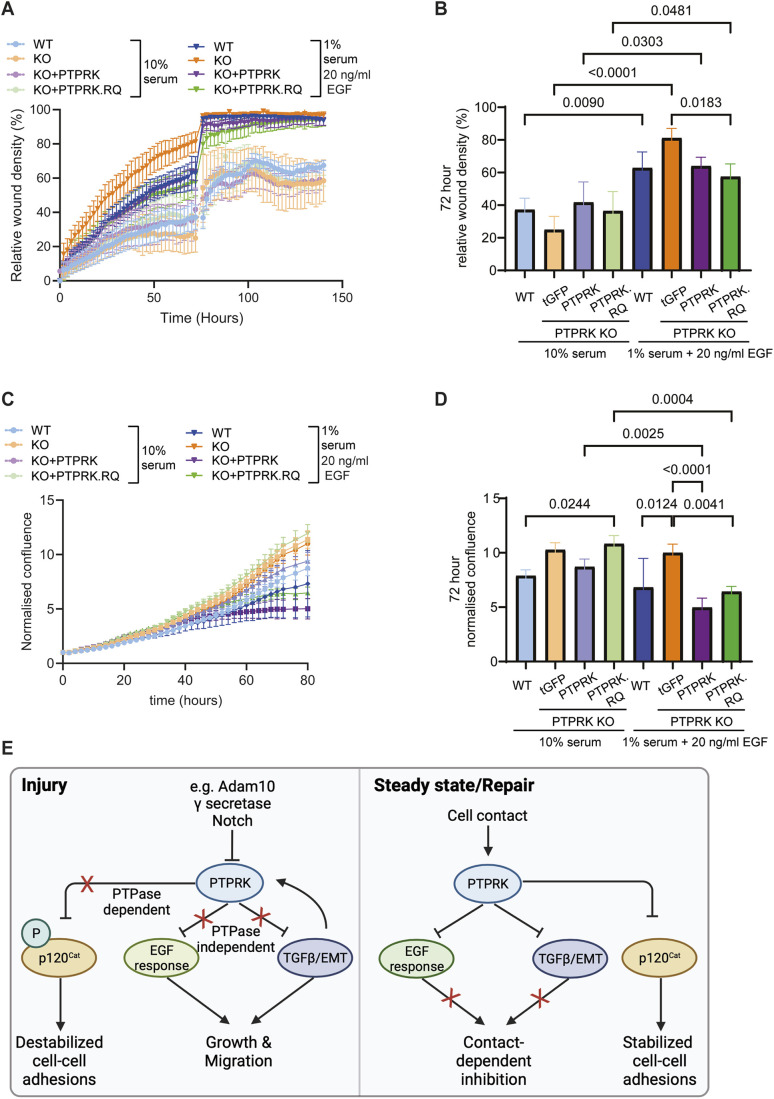
**PTPRK suppresses growth and EMT.** (A) Quantification of relative ratio of the occupied area to the total area of the initial scratched region (wound density) over 6-day scratch wound assays using WT HT29 cells or PTPRK KO HT29 cells with the indicated PTPRK constructs. Data points are plotted for 2 h intervals. All cells were cultured in the presence of 1 µg/ml doxycycline and the indicated media supplements. Scratch wound closure was tracked using the Incucyte Scratch Wound Analysis software module. Media were replenished after 72 h. Mean±s.d., *n*=4. (B) Relative wound density at 72 h derived from A. Mean±s.d., *n*=4. Ordinary one-way ANOVA with Tukey's multiple comparisons test. Comparisons shown are for the same genoype across treatments and between genotypes for the same treatment. Adjusted *P* values (pAdj) shown for comparisons where pAdj<0.05. (C) Relative confluence values for the indicated HT29 cell lines and culture conditions generated by automated Incucyte image acquisition and analysis. Datapoints are every 6 h over a 96 h period. Mean±s.d., *n*=4. (D) Normalized confluence at 72 h derived from C. Mean±s.d., *n*=4. Ordinary one-way ANOVA with Tukey's multiple comparisons test. Comparisons shown are for the same genoype across treatments and between genotypes for the same treatment. Adjusted *P* values shown for comparisons where pAdj<0.05. (E) Summary diagram of PTPRK function in contact-dependent signalling. P, phosphorylation; PTPase, protein tyrosine phosphatase activity.

EGF can promote cell migration (Xie et al., 1998). Under standard cell culture conditions, we found that PTPRK KO HT29 cells were impaired in collective migration, potentially due to perturbed cell junctions (Fig. 1A). We next asked whether PTPRK KO HT29 cells were more responsive to EGF-induced migration, and whether this was catalysis dependent, using a scratch wound assay. Overall, cells were more migratory when stimulated with EGF than when treated with 10% serum. In the presence of EGF, PTPRK KO cells were more efficient at wound healing than WT cells, and this was rescued completely by expression of PTPRK.RQ (Fig. 6A,B). Under the same conditions, we also monitored cell confluence, a measure of cell spreading and proliferation that could also influence wound healing. In contrast to the scratch wound assay, EGF did not stimulate an overall increase in confluence compared to the effects of treatment with 10% serum (Fig. 6C,D). However, we once again saw that in the presence of EGF, PTPRK KO cells were more confluent than WT cells at 72 h, and this effect was rescued by both PTPRK and PTPRK.RQ expression (Fig. 6D). Taken together, our data support a non-catalytic mechanism of EGFR suppression by PTPRK.

## DISCUSSION

In this study, we set out to investigate the function of the homophilic receptor PTPRK in the colon. *Ptprk^−/−^* mice showed no obvious phenotypes; however, when they were subjected to colitis, a role of PTPRK in epithelial integrity or repair was revealed. We also determined that PTPRK is a tumour suppressor both in mouse colon and in a HT29 xenograft model. PTPRK has been proposed to directly dephosphorylate numerous oncoproteins; however, we found that PTPRK can suppress tumour growth and EMT independently of its phosphatase activity. The primary modes of tumour suppression were via regulation of EMT, cell adhesion and growth factor signalling, as determined by phosphotyrosine proteomics and RNA-seq analysis.

These data support previous observations from forward genetics screens of the putative tumour suppressor function of PTPRK in the colon (March et al., 2011; Starr et al., 2009). PTPRK is a recurrent gene fusion with RSPO3, a Wnt pathway potentiator, particularly in serrated adenoma (Sekine et al., 2016), a tumour type associated with a more mesenchymal phenotype (Fessler et al., 2016). The loss of tumour suppressive function of PTPRK by truncation might therefore add increased selective pressure for this gene fusion beyond hyperactivation of Wnt signalling alone, by promoting EMT. In a previous study, PTPRK–RSPO3 expression in mouse colons was found to lead to hyperplasia, but with a distinct molecular profile when compared to loss of the tumour suppressor APC, indicating a potential role for loss of PTPRK activity (Han et al., 2017).

Although HT29 cells lack SMAD4, PTPRK was found to modulate genes associated with EMT. A recent study has demonstrated that *SMAD4* mutations do not prevent colorectal cancer EMT (Frey et al., 2022). Amongst the differentially expressed EMT genes from that study, we find overlap with PTPRK-regulated genes including *TGFB1*, *COL6A1*, *GPX3* and *KLK10*, as well as genes encoding LSR and PKP2, both of which are hyperphosphorylated in PTPRK KO tumours. However, none of the canonical EMT transcription factors –SNAIL (also known as SNAI1), ZEB1, ZEB2, TWIST1 and TWIST2 – were identified. PTPRK has previously been predicted to regulate a hybrid EMT state (Selvaggio et al., 2020), which could be optimal for tumour metastasis (Pastushenko and Blanpain, 2019). This idea is consistent with the increased incidences of invasion that we observed in the *Ptprk* KO mouse model of colorectal cancer. Our data suggest that PTPRK suppresses EMT at the protein and phosphorylation level, as well as via transcriptional regulation of *TGFB1*. *PTPRK* itself is an established TGFβ target gene in several cell types (Stanford et al., 2016; Wang et al., 2005; Xu et al., 2015). We found that TGFβ1 only modestly induced expression of *Ptprk* in intestinal organoid datasets. Furthermore, the Notch1 ICD suppresses *Ptprk* expression, which is contrary to a previous report of increased PTPRK expression induced by the Notch ligand Jag in keratinocytes (Xu et al., 2015), and could indicate that tissue-specific regulation by Notch promotes metastasis (Jackstadt et al., 2019) via TGFβ and is also important for recovery from DSS-induced colitis (Okamoto et al., 2009). Additionally, we found a potential link between PTPRK phosphatase activity and metabolic signalling, which has recently been investigated in the liver (Eduardo et al., 2023 preprint).

In response to injury, epithelia must undergo a partial EMT (Stone et al., 2016) and increase their responsiveness to growth factors via reversal of contact inhibition (Mendonsa et al., 2018). PTPRK is ideally placed as a contact sensor. We speculate that injury responses require PTPRK downregulation to promote partial EMT and increase sensitivity to growth factors. We propose a model of temporary inactivation of PTPRK to facilitate epithelial repair (Fig. 6E). Inhibition of PTPRK activity could be controlled by damage signalling, including oxidation (Xu et al., 2006), Notch activation or directly by upregulation of proteases such as ADAM10 and γ-secretase (Anders et al., 2006; Kuhn et al., 2016; Sachs et al., 2021). As a result of losing PTPRK from the cell surface (e.g. at the new leading edge), pro-mesenchymal signalling would be derepressed, and cell junctions destabilized due to p120^Cat^ hyperphosphorylation, enabling cells to adopt a hybrid EMT state. As the injury resolves and damage signalling subsides, PTPRK protein would sense new cell contacts forming as the epithelium repairs. Through its accumulation at nascent cell contacts, PTPRK would then suppress growth factor responsiveness and EMT signalling and stabilize cell junctions, supporting a reversion to an epithelial state. Cancer has been likened to a ‘wound that will not heal’ (Byun and Gardner, 2013), and in this way PTPRK KO cells are in a constant state of wound healing.

The direct dephosphorylation of RTKs by RPTPs is a widely held notion in the field (Östman et al., 2006), yet our data clearly demonstrate that PTPRK can non-catalytically suppress growth factor receptor phosphorylation following ligand stimulation. We chose to use the R1095Q mutant of PTPRK because it does not ‘trap’ interactors, unlike catalytic cysteine mutants (Fig. 3A). This could explain the difference between our results and those previously reported where cysteine mutants were used in rescue experiments (Chabot et al., 2009; Joshi et al., 2023; Yao et al., 2017). PTPRK has been implicated in the direct dephosphorylation of numerous oncogenic substrates, including β-catenin and STAT3, yet we could not find evidence in support of this in our previous study (Fearnley et al., 2019). Here, we also identified hyperphosphorylated proteins in PTPRK KO cells that do not appear to be direct substrates, such as ARHGAP35 (a p190RhoGAP) and PKCδ (Fig. S6A). Non-catalytic functions of PTP domains have been reported. For example, PTPRB can regulate cell adhesion in endothelial cells independently of its catalytic activity via an interaction with ARHGEF2 (also known as GEF-H1; Juettner et al., 2019), which is also an interactor of PTPRK (Fearnley et al., 2019). Notably, in that study a PTPRB-WPD motif inactivating mutant was used, which behaves differently to cysteine mutant traps (Fearnley et al., 2019; Flint et al., 1997). Thus, our study supports non-catalytic modulation of signalling by PTPRK.

How might non-catalytic modulation of signalling by PTPRK be achieved? A previous study has suggested that PTPRK knockdown increases the levels of phosphorylated EGFR in endosomes following EGF stimulation (Villaseñor et al., 2015). This could be consistent with a role for PTPRK in receptor internalization and trafficking, or effects on a scaffold (for example, an SH2 domain-containing protein) that protects EGFR from dephosphorylation. The PTPRK constructs used in our study possess the PTPRK D2 domain, which can mediate protein–protein interactions (Hay et al., 2022a). Our previous study and a BioID screening approach have revealed an extensive set of RPTP-associated proteins (Fearnley et al., 2019; St-Denis et al., 2016). This raises the possibility of PTPRK protein–protein interactions that mediate repression of growth factor and EMT signalling. The most enriched PTPRK D2 domain interactor identified was FAM83B, which is a scaffold for casein kinase 1 (CK1) isoforms that localizes at cell contacts (Fulcher et al., 2018). FAM83B binding to PTPRK was unaffected by D1 domain mutations (Fig. 3A). FAM83B is a paralogue of FAM83G, which has been implicated in both Wnt and bone morphogenetic protein signalling (Bozatzi et al., 2018), by promoting CK1-mediatied phosphorylation of β-catenin and SMAD1, respectively. Furthermore, FAM83B is also a positive regulator of EGFR signalling (Cipriano et al., 2014). Recently another paralogue, FAM83H, has been identified as a regulator of cell junctions (White et al., 2023). Whether PTPRK inhibits FAM83B regulation of EMT, Wnt signalling or growth factor signalling is an important next question. The non-receptor phosphatase PTPN14, another PTPRK D2 domain interactor (Fearnley et al., 2019), functions as a tumour suppressor and has been suggested to regulate Rab5 to Rab7 endosomal maturation, impacting on EGFR signalling. This is thought to be regulated by PTPN14 control of PKCδ (Lonic et al., 2021), which we found to be hyperphosphorylated in PTPRK KO xenografts. Thus, PTPRK might bind and activate PTPN14 to modulate endosomal maturation and downregulate EGFR signalling. Further investigation of the non-catalytic functions of PTPRK is required to elucidate its mechanisms of tumour suppression.

## MATERIALS AND METHODS

### Cells and cell culture

HT-29 cells were purchased from ATCC (Table S4). Cells were maintained in 75 cm^2^ vented tissue culture flasks in a 37°C humidified 5% CO_2_ ventilator and passaged using trypsin-EDTA solution (Sigma-Aldrich) prior to reaching confluence every 2–4 days. HT-29 cells were grown in DMEM (Thermo Fisher Scientific) containing 10% (v/v) foetal bovine serum (FBS; Sigma-Aldrich) and 2 mM L-glutamine (Sigma-Aldrich). HT-29 cells with stably integrated lentiviral vectors were cultured in 1 μg/ml puromycin (Gibco, Thermo Fisher Scientific). MCF10A PTPRK KO and parental cells were generated previously (Fearnley et al., 2019) and were grown in 1:1 DMEM (Thermo Fisher Scientific):Ham's F12 (Sigma-Aldrich), supplemented with 5% (v/v) horse serum (Thermo Fisher Scientific), 20 ng/ml EGF (Peprotech), 0.5 mg/ml hydrocortisone (Sigma-Aldrich), 100 ng/ml cholera toxin (Sigma-Aldrich) and 10 mg/ml insulin (Sigma-Aldrich) (Debnath et al., 2003). DLD1 cells were obtained from the Cook lab at the Babraham Institute and were cultured in RPMI (Thermo Fisher Scientific) with 10% FBS and 2 mM L-glutamine. All cell lines were routinely mycoplasma tested using a MycoProbe Mycoplasma Detection Kit (R&D Systems) or a MycoAlert PLUS Mycoplasma Detection Kit (Lonza).

### CRISPR/Cas9 genome editing

Oligonucleotides for single guide RNAs targeting exons 1 and 2 of PTPRK cloned into pSp.Cas9.(BB).eGFP were used as previously described (Fearnley et al., 2019). HT-29 cells were transfected using Lipofectamine LTX with PLUS Reagent as per the manufacturer's instructions (Thermo Fisher Scientific). After 72 h, HT29 cells underwent negative selection by flow cytometry using an anti-PTPRK monoclonal antibody (Table S4). Sorted cells were expanded, and PTPRK protein levels were assessed by immunoblotting (Table S4). DLD1 cells were transfected with plasmids using Genejuice (EMD Chemicals Inc), as per the manufacturer's instructions. After 48 h, eGFP-positive cells were single-cell sorted using flow cytometry. Clones were expanded, and PTPRK protein levels assessed by immunoblotting (Table S4). Following confirmation of PTPRK KO, three clones were combined.

### Plasmids and constructs

Amino acid (aa) numbering is based on the following sequences: PTPRK, UniProt ID Q15262-3; PTPRF, Uniprot ID P10586-1. For inducible lentiviral expression in mammalian cells, PTPRK cDNA was previously subcloned in-frame into pCW57.GFP.2A.MCS (Fearnley et al., 2019). For bacterial expression, human coding sequence corresponding to the PTPRF ICD (aa 1328–1907) was subcloned into a modified pET-15b bacterial expression vector in frame encoding an N-terminal His.TEV.AviTag (Fearnley et al., 2019). Generation of PTPRK ICD constructs was previously described (Fearnley et al., 2019). All point mutations were introduced by PCR using either Q5 High-Fidelity DNA (New England Biolabs, UK) or Phusion Hot Start II DNA (Thermo Fisher Scientific) polymerases as per manufacturer's protocol.

### SDS-PAGE and immunoblotting

Protein or cell lysate samples were resuspended in an appropriate volume of 5× SDS-PAGE sample buffer [0.25 M Tris-HCl pH 6.8, 10% (w/v) SDS, 20% (v/v) glycerol, 0.1% (w/v) Bromophenol Blue, 10% (v/v) β-mercaptoethanol] and incubated at 92°C for 10 min. Samples were run on a 10% (v/v) SDS-polyacrylamide resolving gel with a 5% (v/v) SDS-PAGE stacking gel and subjected to electrophoresis at 125 V for 1.5 h in 25 mM Tris, 190 mM glycine and 0.1% (w/v) SDS. Proteins were transferred onto 0.2 µm reinforced nitrocellulose membranes (GE Healthcare) at 300 mA for 3 h at 4°C in 25 mM Tris, 190 mM glycine and 20% (v/v) methanol. Membranes were briefly rinsed in TBS-T [20 mM Tris-HCl pH 7.6, 137 mM NaCl, 0.1% (v/v) Tween-20] prior to incubation for 30 min in TBS-T containing 5% (w/v) skimmed milk to block non-specific antibody binding. The blocking solution was removed, and membranes rinsed in TBS-T prior to primary antibody incubation (overnight at 4°C). Membranes were then subjected to 3×10 min washes in TBS-T, prior to incubation with HRP-conjugated species-specific anti-IgG antibodies (1 h at room temperature). Membranes were then subjected to 3×10 min washes in TBS-T, prior to being incubated with combined EZ-ECL solution (Cytiva) and imaged using a G:BOX Chemi XX6 (Syngene). See Table S4 for antibody details. Uncropped immunoblots are presented in Fig. S7.

### Cell proliferation assay

Cell proliferation was measured using the MTS-based CellTiter 96 AQueous One Solution Cell Proliferation kit (Promega G3582). Briefly, 20,000 or 40,000 MCF10A cells were seeded in triplicate in a 96-well plate with a medium-only control. After 72 h, the MTS assay was carried out as per the manufacturer's instructions. The absorbance was measured at 490 nm using a 96-well plate reader (SpectraMax, M5, Molecular Devices, UK) after a 30 min incubation period with the CellTiter 96 AQueous One Solution Reagent.

### Recombinant GST-tagged protein production

pGEX-PAK-CRIB (Addgene #12217; deposited by Dr Jonathan Chernoff; Table S4) was transformed into *E. coli* Rosetta BL21 (DE3) cells (Sigma-Aldrich) and grown in a 50 ml starter culture of terrific broth (Melford) with ampicillin (100 μg/ml) overnight at 37°C. The following day, the culture was expanded into 500 ml terrific broth with ampicillin (100 μg/ml). Protein expression was induced with 0.1 mM isopropyl-thio-b-D-galactopyranoside (IPTG; Generon) for 2 h. Bacteria were pelleted at 1500 ***g*** for 30 min at 4°C. Pellets were resuspended in 15 ml of ice-cold bacterial lysis buffer [20% sucrose, 10% glycerol, 50 mM Tris-HCl (pH 8), 0.2 mM Na_2_S_2_O_5_, 2 mM MgCl_2_, protease inhibitor (Roche)] and lysed by probe sonication (Misonix S-3000 probe sonicator) at an amplitude of 6 for six cycles of 10 s with 10 s pauses on ice in between each sonication.

Lysates were centrifuged at 40,000 ***g*** for 30 min at 4°C, and supernatants were added to 500 μl of pre-washed glutathione sepharose beads (GE Healthcare) followed by 1 h incubation at 4°C with end-over-end rotation. After incubation, the beads were washed twice with bacterial lysis buffer and twice with GST-FISH buffer [50 mM Tris-HCl (pH 7.4), 10% glycerol, 100 mM NaCl, 1% NP40, 2 mM MgCl, protease inhibitor (Roche)], and expression of the GST-tagged Rac-binding domain was visualized on Coomassie-stained SDS-PAGE gels alongside bovine serum albumin (BSA) standards.

### Recombinant biotinylated protein production

*E. coli* BL21 (DE3) Rosetta cells transformed with the relevant expression construct (Table S4) were cultured at 30°C and 220 rpm in 1 l of 2×TY medium (Sigma-Aldrich) containing 50 mg/ml carbenicillin and 34 mg/ml chloramphenicol until reaching OD600 0.6–0.7. Cultures were then transferred to 20°C and 220 rpm and allowed to equilibrate prior to the addition of 1 mM IPTG (Generon) and 200 mM of D-biotin (Sigma-Aldrich). Cells were harvested after 20 h by centrifugation at 4000 ***g*** for 30 min, and bacterial pellets were stored at −20°C until required. Prior to lysis, cells were subjected to one round of freeze–thaw. Cells were lysed in purification buffer [50 mM HEPES pH 7.5, 500 mM NaCl, 5% (v/v) glycerol and 0.5 mM TCEP] containing EDTA-free protease inhibitor (Roche) using a Constant Systems cell disruptor, and the cell extract was clarified via centrifugation at 40,000 ***g*** for 30 min at 4°C. The supernatant was removed and incubated with 0.5 ml of Ni-NTA agarose (Qiagen) for 1 h at 4°C. Ni-NTA agarose was then pelleted via centrifugation at 500 ***g*** for 5 min at 4°C and packed into a gravity flow column. Ni-NTA agarose was then washed with ten volumes of purification buffer containing 5 mM imidazole, followed by 20 volumes of purification buffer containing 20 mM imidazole; prior to elution in purification buffer containing 250 mM imidazole. The eluted protein was then subjected to size exclusion chromatography (SEC) using a Superdex 200 16/600 column (GE Healthcare Life Sciences, Thermo Fisher Scientific). Columns were equilibrated in SEC buffer [50 mM HEPES pH 7.5, 150 mM NaCl, 5% (v/v) glycerol, 5 mM DTT]. Protein was concentrated to 2–10 mg/ml using an Ultracel-10K regenerated cellulose centrifugal filter (Merck Millipore) prior to snap-freezing and storage at −80°C until required. The purified protein was assessed for purity by SDS-PAGE and staining with InstantBlue (Expedeon, UK).

To assess biotinylation, 10 μg of *in vivo* biotinylated protein was solubilized in an appropriate volume of 5× SDS loading buffer and heated to 95°C for 5 min. Samples were allowed to cool to room temperature prior to addition of a threefold molar excess of streptavidin (ACRO Biosystems). Samples were incubated for 10 min at room temperature, then immediately analysed by SDS-PAGE followed by Coomassie staining. Approximate levels of biotinylation were calculated by 2D densitometry of protein band depletion upon addition of streptavidin using Fiji (Schindelin et al., 2012).

### pNPP phosphatase activity assay

Recombinant PTP domains were made up to 500 μl in assay buffer [50 mM Tris-HCl, pH 7.4, 150 mM NaCl, 5% (v/v) glycerol, 5 mM DTT] at 0.6 μM. PTP domains and 20 mM p-nitrophenyl phosphate (pNPP) substrate (in assay buffer) were equilibrated to 30°C for 15 min in an orbital shaking heat block at 500 rpm. To initiate reactions, 500 μl of 20 mM pNPP substrate was added to PTP-containing tubes (0.3 μM PTP and 10 mM pNPP final concentrations). Reactions were carried out for 120 min at 30°C in an orbital shaking heat block at 500 rpm. At each time point (0, 2, 5, 10, 15, 30, 60, 90 and 120 min), 100 μl of the total reaction was transferred to a 96-well microplate well containing 50 μl 0.58 M NaOH, terminating the reaction. After the final time point, absorbance of each sample was measured at 405 nm in a Spectramax M5 plate reader (Molecular Devices). Product formation was calculated by interpolation of absorbance values using a 4-nitrophenol standard curve of known concentration.

### GST pull-down assays

Cells were seeded at a density of 3×10^6^ cells in a 10 cm^2^ dish for 72 h. Dishes were placed on ice and the medium was aspirated. Next, 1.2 ml of cold GST-FISH buffer was added, and cells were scraped and collected in pre-cooled 1.5 ml Eppendorf tubes. Samples were incubated on ice for 5 min with intermittent vortexing. Samples were then centrifuged at 12,000 ***g*** for 5 min at 4°C. A 75 μl volume was collected from the supernatant to run as the total lysate control. The remaining sample was added to pre-aliquoted beads and incubated for 15 min at 4°C with end-over-end rotation. The beads were then washed three times in 1 ml cold GST-FISH buffer. 4× SDS-PAGE loading buffer (25 μl) was added to the beads and total lysate controls before samples were boiled for 5 min. 1% total cell lysate and pull-down samples were resolved by SDS-PAGE (precast 4–12% gels, Thermo Fisher Scientific) and transferred to nitrocellulose membranes. Bound Rac1 was detected by immunoblotting using an antibody against Rac1 (Millipore; Table S4).

### Pervanadate treatment

A fresh 44.2 mM pervanadate stock solution was generated based on a previously described method (Huyer et al., 1997). Briefly, 100 μl of 100 mM sodium orthovanadate (Alfa Aesar) was combined with 103 μl 0.49 M H_2_O_2_ (Sigma-Aldrich) in 20 mM HEPES, pH 7.3, mixed by gentle inversion and incubated at room temperature for 5 min and quenched by addition of 23 μl of 0.5 mg/ml catalase (Sigma-Aldrich) in 50 mM potassium phosphate, followed by mixing by gentle inversion. Confluent MCF10A cells were treated with 100 μM pervanadate for 30 min in a 37°C, 5% CO_2_ incubator, then transferred to ice and washed twice with ice-cold PBS. Each dish was lysed in 600 μl lysis buffer [50 mM Tris–HCl, pH 7.4, 150 mM NaCl, 10% (v/v) glycerol, 1% (v/v) Triton X-100, 1 mM EDTA, 5 mM iodoacetamide (IAA), 1 mM sodium orthovanadate, 10 mM NaF, 1× EDTA-free protease inhibitor (Roche)] on ice, with periodic agitation, in the dark at 4°C for 30 min. Lysates were scraped, collected into 1.5 ml tubes, and treated with 10 mM DTT on ice for 15 min. Lysates were cleared by centrifugation (14,000 ***g***, 15 min, 4°C). Supernatants were then collected, snap frozen in liquid nitrogen and stored at −80°C until use.

### Streptavidin pull-down assays

For each experimental condition, 167 µl of streptavidin-coated magnetic bead suspension (4 mg/ml; New England Biolabs) was washed thrice with resuspension with 1 ml of ice-cold 150 mM NaCl wash buffer [20 mM Tris-HCl pH 7.4, 150 mM NaCl, 10% (v/v) glycerol, 1% (v/v) Triton X-100, 1 mM EDTA pH 8.0]. 50 µg of biotinylated His.TEV.Avi.PTPRK domains were conjugated to 167 µl of pre-washed streptavidin-coated magnetic beads suspension in 1 ml of ice-cold 5% (w/v) BSA in 150 mM NaCl wash buffer containing 1× EDTA-free protease inhibitors (Roche) at 4°C for 1 h on a rotator. A beads-only control was treated identically. Simultaneously, 1 ml of 1 mg/ml per sample of freshly thawed pervanadate-treated cell lysate was then pre-cleared with streptavidin-coated magnetic beads (167 µl of bead suspension per ml of lysate) at 4°C for 1 h on a rotator. Blocked, conjugated PTPRK beads were then briefly spun, transferred onto a magnetic stand and washed three times with 1 ml of ice-cold 150 mM NaCl wash buffer, prior to incubation with 1 ml of 1 mg/ml pre-cleared pervanadate-treated lysate at 4°C on for 1.5 h on a rotator. Beads were then washed thrice with resuspension in 1 ml ice-cold 150 mM NaCl wash buffer containing additional 10 mM DTT, including a brief spin and separation by magnet. Next, beads were washed thrice with resuspension in 1 ml ice-cold 500 mM NaCl wash buffer [20 mM Tris-HCl pH 7.4, 150 mM NaCl, 10% (v/v) glycerol, 1% (v/v) Triton X-100, 1 mM EDTA pH 8.0 with additional 10 mM DTT]. Finally, beads were washed twice with 1 ml ice-cold TBS (20 mM Tris-HCl pH 7.6, 137 mM NaCl). For immunoblot analysis, beads were resuspended in 20 μl of 18% (v/v) formamide, 1 mM EDTA pH 8.0 made up in TBS, incubated at 95°C for 5 min, followed by addition of 30 μl of 5× SDS-PAGE sample buffer containing 25 mM biotin and incubated at 95°C for 10 min. After a brief spin, beads were separated by magnet and supernatants subjected to SDS-PAGE and immunoblotting.

### In-lysate dephosphorylation assays

Recombinant PTP domains (90 nM final concentration) were added to 50 µl (125 µg) of pervanadate-treated MCF10A lysate in a total volume of 200 µl wash buffer [50 mM Tris-HCl, pH 7.4, 150 mM NaCl, 10% (v/v) glycerol, 1% (v/v) Triton X-100]. Reactions were incubated with end-over-end rotation for 30 min at 4°C. 4 µl 10% (w/v) SDS [0.4% (w/v) SDS final] was added to 100 µl of the sample to stop the reaction, then samples were briefly vortexed and placed on ice for 10 min. 33 µl 5× SDS-PAGE sample loading buffer was added and samples incubated for 10 min at 95°C. Samples were stored at −20°C prior to SDS-PAGE and immunoblot analysis.

### Lentivirus production and infection

1.5×10^7^ HEK293T cells (as used previously in Fearnley et al., 2019) were seeded in 12 ml of complete growth medium per 15 cm^2^ dish (two dishes per lentivirus) and incubated for 24 h at 37°C with 5% CO_2_. Each 15 cm^2^ dish was then transfected with either 6 μg of pCW57.GFP.2A or pCW57.GFP.2A.PTPRK (Table S4), 12 μg of the psPAX2 packing plasmid (Addgene #12260, deposited by Didier Trono,) and 3 μg of the pMD2.G envelope plasmid (Addgene #12259, deposited by Didier Trono) using the GeneJuice transfection reagent (Merck Millipore, UK) as per manufacturer's instructions. After 24 h, the medium was then replaced with 16 ml complete growth medium. At 48–72 h post-transfection, culture medium was collected and filtered through a 0.45 μm mixed cellulose esters membrane (Sartorius). Viral particles were pelleted via ultracentrifugation at 100,000 ***g*** for 1.5 h at 4°C and resuspended in 600 μl of OptiMEM (Thermo Fisher Scientific) or using Lenti-X concentrator as per manufacturer's instructions (Takara Bio). Lentivirus was aliquoted and stored at −80°C until required.

For lentiviral infections, 1.6×10^5^ HT-29 cells were seeded per well of a six-well plate in 900 μl of growth medium, prior to the drop-wise addition of 100 μl concentrated lentivirus. After 30 min at room temperature, cells were returned to the incubator. 72 h later, cells were reseeded in 1 μg/ml puromycin (Thermo Fisher Scientific) selection medium. The cells were then sorted based on tGFP expression.

### Scratch wound assay

For quantification, cells were seeded at 200,000 cells per well in a 96-well ImageLock tissue culture plate (Essen BioScience). Cells were incubated overnight. The following day, a WoundMaker (Essen Bioscience) was used to make a single scratch in every well of the plate. The wells were washed twice with medium to remove debris. 100 µl of medium was then added to every well before the plate was placed inside the IncuCyte and the IncuCyte S3 Live-Cell Analysis System was used to take images every 2 h for 6 days using the scratch wound mode. The medium was changed every 3 days. The IncuCyte scratch wound analysis software was then used to analyse the relative wound density of each scratch.

For immunofluorescence, 200,000 cells per condition were plated in a 96-well plate in quadruplicate with 200 µl of medium per well. After 24 h, the wells were scratched using a multichannel pipette and washed once with PBS before adding 200 µl of medium per well (containing 1 µg/ml doxycycline). The scratches were then imaged manually. The medium was changed on day 4 of the experiment. On day 7, the scratches were imaged manually. Quantification of the area of the scratch wound was carried out using ImageJ software. For quantification of γ-tubulin polarization, ‘correct’ polarization was defined by placing a 120° sector facing the wound for each cell at the leading edge.

### Quantifying cell migration into scratch wounds

Cells were seeded and scratched as described above. The IncuCyte S3 Live-Cell Analysis System was used to image the scratch wounds every 2 h for 48 h. Using ImageJ, ten WT or PTPRK KO cells on the leading edge of the scratch wound were selected, and they were tracked over 48 h using the Manual Tracking plug-in. This gave the *x* and *y* coordinates of the migrating cells on the leading edge, which could then be plotted to create a migration plot. The coordinates of 30 cells from three independent repeats were plotted on a single graph.

### Confluence assay

HT29 cells were seeded at 10,000 cells per well in a 96-well tissue culture plate, with four replicates per genotype. Cells were incubated overnight, and the plate was placed inside the IncuCyte. Images were taken from each well every 2 h. The IncuCyte confluence analysis software was then used to analyse each image. The data were normalized by division to the start value.

### Immunofluorescence

Cells were grown with or without doxycycline on coverslips in six-well plates, then fixed 72 h after the addition of doxycycline using 4% paraformaldehyde in PBS for 10 min. The cells were then permeabilized in 0.5% Triton X-100 and 3% BSA in PBS for 2 min and blocked in 0.2% Triton X-100 and 3% BSA in PBS (blocking buffer) for 1 h. Primary antibodies in blocking buffer were incubated for 1 h and secondary antibodies were incubated for 45 min. Antibodies used for immunofluorescence staining are shown in Table S4. The imaging was done with a Zeiss LSM 780 confocal microscope and a 63× objective.

### Co-culture immunofluorescence

500,000 WT and KO cells were mixed and plated together on a coverslip. Cells were cultured for 3 days before fixation and immunostaining following the protocol described above. Slides were imaged using a Leica Stellaris confocal microscope with a 40× oil immersion objective. Intensity was measured using ImageJ software. An area of the same size was used to calculate the mean antibody signal intensity of p120^Cat^ at the membrane between two WT or two KO cells. The value was normalized dividing it by the value of a background measurement. Five different measurements of intensity at WT-WT and KO-KO junctions were taken per image. The average of each value was used. Six different images were analysed.

### Generation of PTPRK deletion in mice using CRISPR/Cas9

Mice were generated according to protocols approved by the Genentech Institutional Animal Care and Use Committee. The *Ptprk* null allele was generated using CRISPR/Cas9 and zygotes from C57BL/6N, essentially as described previously (Anderson et al., 2018). The sequence of the sgRNA used was 5′-ACTTCTCCCAAACTCGCCA-3′, which is located in *Ptprk* exon 1. It corresponds to genomic coordinates (GRCm38/mm10) chr10:28,075,063-28,075,081. The 22 bp deletion corresponds to chr10:28,075,077–28,075,098, and the deleted region includes the Kozak sequence and initiation ATG. Mice were imported to the UK as cryopreserved sperm and subsequently rederived. Breeding and experiments were performed according to protocols previously approved by the UK Home Office (PPL: P1D1004EC).

Mice were genotyped using PCR with specific PTPRK primers that amplify a 234 bp region of exon 1 containing the 22 bp deletion: forward, 5′-CAAAGCTGCTTGAAACTTCT-3′; reverse, 5′-AAGACTGTGGACAGACAC-3′. The Phire II tissue direct PCR kit (Thermo Fisher Scientific, F170S) was used to extract and amplify DNA, which was then run on a 3% agarose gel to identify a 22 bp shift. PTPRK protein knockout was confirmed using immunoblotting.

### Immunophenotyping and cytotoxic T lymphocyte cytotoxicity assays

Immunophenotyping and cytotoxic T lymphocyte (CTL) cytotoxicity were measured as previously described (Abeler-Dörner et al., 2020) using the CytoTox 96 Non-Radioactive Cytotoxicity Assay (Promega, G1780). Briefly, splenocytes were incubated in the dark at room temperature for 30 min with PE-conjugated rat anti-mouse CD4 and APC-conjugated rat anti-CD8 (Table S4) followed by a wash in 1% FBS in sterile DPBS (Gibco). Following resuspension cells were analysed using a FACSCalibur flow cytometer (BD Biosciences). Data were analysed using FlowJo version 7.8.

For cytotoxicity assays, 0.1×10^6^ CD4^+^/CD8^+^ cells were seeded in wells of a round-bottom 96-well plate, resuspended in phenol-red free RPMI 1640 (Life Technologies) supplemented with 2% heat-inactivated FBS and 1% penicillin-streptomycin (Sigma-Aldrich). 0.01×10^6^ target cells (P815; ATCC) and 0.5 µg/ml anti-mouse CD3e (Sigma-Aldrich) were seeded into a twofold serial dilution of effector cells. Cells were incubated at 37°C for 3 h. Lysis buffer (Promega) was added to positive control wells and incubated for 45 min. Following centrifugation at 200 ***g*** for 2 min, 50 µl supernatants were transferred to wells of a flat-bottom 96-well plate. 50 µl substrate mix (Promega) was added per well to the supernatants for 30 min in the dark at room temperature before acquisition on a SpectraMax plate reader (Molecular Devices) at the absorbance wavelength of 490 nm. Percentage lysis was calculated as follows: (((CTL+P815+anti-CD3e)−(CTL+P815))/positive control)×100.

### DSS-induced colitis

Male mice, aged between 2 and 3 months, were treated with DSS dissolved in drinking water at 2% (w/v) for 7 days. The mice were then placed back onto untreated drinking water for the remaining 7 days. Experimental endpoints were defined as: weight loss of >15%, or sustained inactivity (2–3 h) and hunched posture (or vocalizing, or ruffled and wet coat, or closed eyes), or rectal prolapse.

### AOM–DSS-induced colorectal tumours

Male mice, aged between 2 and 3 months, were administered with an intraperitoneal injection of AOM (10 mg/kg) that had been diluted in sterile PBS. The mice were then given 3 days recovery before the first DSS cycle started. The mice were treated with DSS in three cycles with 2 week breaks between each cycle. Cycle 1, 1.5% DSS for 5 days; cycle 2, 1% DSS for 4 days; cycle 3, 1% DSS for 1 day. At 2 weeks after the final cycle, the mice were culled, and entire lengths of colon were removed and placed in 10% neutral buffered formalin.

### Tissue processing and sectioning

Tissues were dehydrated by placing in the following ethanol concentrations for sequential 1 h incubations: 30%, 50%, 70%, 100%, 100%. Samples were then left overnight at 4°C in 100% ethanol. Next, tissues were incubated in xylene for 3×1 h washes. They were then placed in a wax wash in a 60°C hybridization oven for 1 h, and the wax wash repeated three times. The tissues were then embedded in paraffin wax. Paraffin-embedded tissues were sectioned using a microtome into 5 µm sections onto superfrost plus slides (Thermo Fisher Scientific).

### Haematoxylin and Eosin staining

Sections on slides were deparaffinized by successive changes in xylene, 95% (v/v) ethanol and finally 70% alcohol. The sections were stained for 5 min in Harris Haematoxylin solution (Merck, HHS32), differentiated in 1% acid alcohol, then blued using Scott's tap water substitute (Sigma-Aldrich, S5134). Lastly, the slides were counterstained in Eosin-Y solution (Cell path, RBC-0100-00A) for 30 s. The sections were then dehydrated through 95% alcohol, absolute alcohol and xylene. They were mounted using DPX mountant for histology (Merck, 06522). Images were obtained using a Zeiss AxioImager Z2 upright wide-field microscope with a 10× objective for Fig. S2G and a Nikon Ti2 wide-field microscope with a 20× objective for Fig. 2G.

### Immunofluorescence staining of tissues

Samples were fixed, sectioned and deparaffinized as described above, followed by antigen retrieval using a pressure cooker for heat-induced epitope retrieval (https://www.abcam.com/protocols/ihc-antigen-retrieval-protocol). The slides were then stained for immunofluorescence (see Immunofluorescence section above). Slides were imaged using the Zeiss AxioImager Z2 upright wide-field microscope with a 63× objective.

### Tumour xenografts

Four HT-29 cells lines (WT, PTPRK KO with tGFP, PTPRK KO with PTPRK rescue construct, and PTPRK KO with PTPRK.RQ mutant) were harvested following *in vitro* culture and subcutaneously injected at 1×10^6^ per mouse in 100 µl of PBS and Matrigel in a 1:1 ratio into the right flank of 8-week-old NU(NCr)-Foxn1nu female mice (Charles River; Table S4). Eight mice were used per cell line. Mice received 1.5 mg/ml of doxycycline dissolved in their drinking water containing 5% sucrose. The four different genotypes were co-housed to ensure equal access to food and water. Body weights and tumour diameters were measured three times per week. The endpoint was reached once the tumour grew to 12 mm mean diameter, as determined by the formula (length+width)/2.

### HT29 sphere cultures

Agarose (Sigma-Aldrich) was diluted to 1.5% in DMEM (Thermo Fisher Scientific) and heated in the microwave until fully dissolved. The solution was then plated into a flat-bottomed 96-well plate (50 µl/well) and allowed to cool for 20 min. 1.5×10^3^ HT29 cells in 200 µl of full medium were plated on top and grown to form spheres for 7 days with a medium change every 3–4 days. Sphere growth was tracked using an IncuCyte S3 Live-Cell Analysis System (spheroid software module). Images were taken from day 4 and analysed using ImageJ to calculate circularity of individual spheroids using their area and perimeter. Average circularity was calculated from ≥15 spheres per WT or PTPRK KO genotype, and the experiment was repeated three times.

### Invasion assay

Cells were cultured in the presence of 1 µg/ml Matrigel for 4 days prior to experiments. Matrigel (Corning) was diluted 33% in coating buffer (1 M Tris-HCl pH 8.8 with 0.7% NaCl). 8 mm inserts (Thermo Fisher Scientific) were then coated with 100 µl of diluted Matrigel (Corning). 200,000 HT29 cells were seeded on top of the Matrigel in the upper chamber of each well of a 24-well Falcon TC companion (Corning) plate in 500 µl serum-free medium, and the bottom chamber was filled with 750 µl culture medium. The cells were then incubated for 48 h. Following this, the invasive cancer cells were fixed on the inserts with 4% paraformaldehyde (Thermo Fisher Scientific) for 2 min then washed twice with PBS and then permeabilized in methanol for 20 min. After a further two washes in PBS, the cells were stained with 0.2% Crystal Violet (diluted in 20% methanol in sterile water) for 30 min. Finally, the inserts were washed twice in PBS and non-invasive cells were scraped off using a cotton bud. Images were captured using Zeiss AxioImager Z2 upright wide-field microscope with a 4× objective.

### Preparation and sequencing of RNA libraries

Tumours were ground to a fine powder in liquid nitrogen using a mortar and pestle, as previously described (Robertson and Leek, 2006). Four or five replicates were used for each cell line. RNA was extracted using RNeasy plus minikit according to the manufacturer's instructions (Invitrogen). RNA quality and quantity was assessed using a Bioanalyzer 2100, and all samples presented RNA integrity number (RIN) higher than 6.8. Library preparation was performed by Novogene. In brief, after quality control, mRNA was poly(A) enriched using oligo(dT) beads. mRNA was fragmented randomly by adding fragmentation buffer, then cDNA synthesized using mRNA template and random hexamer primers, after which a custom second-strand synthesis buffer (Illumina), dNTPs, RNase H and DNA polymerase I were added to initiate the second-strand synthesis. After a series of terminal repair, a ligation and sequencing adaptor ligation, the double-stranded cDNA library was completed through size selection and PCR enrichment. The experiment was paired end with 20–50 million 150 bp reads using PE150 on NovaSeq 6000.

### RNA-seq read alignments and differential expression analysis

Raw FASTQ reads were trimmed with trim_galore v0.6.6 (https://www.bioinformatics.babraham.ac.uk/projects/trim_galore/) using default parameters then mapped separately to the GRCh38 human and GRCm38 mouse assemblies using hisat2 v2.3.5.1 (Kim et al., 2019) using options -no-mixed -no-discordant. Mapped reads with a MAPQ<20 were discarded. Samples were quantitated as raw exon overlap counts at the gene level using SeqMonk v1.47.1 (https://www.bioinformatics.babraham.ac.uk/projects/seqmonk/) and the Ensembl v102 gene annotations in non-directional mode. Genes annotated with a lncRNA or antisense biotype were removed, as were genes that were unexpressed in all samples. For tSNE clustering and Intensity Difference statistics the quantifications were converted to log_2_ fragments per million mapped fragments (FPM). Comparisons between conditions were initially made with DESeq2 (Love et al., 2014) using a significance threshold of false discovery rate (FDR)<0.05. DESeq2 hits were further filtered by intersecting with hits from the SeqMonk Intensity Difference filter, run with a cut-off of FDR<0.05. The tSNE profile was generated using default parameters with a perplexity of 5. Differentially expressed gene lists were submitted to EnrichR (Xie et al., 2021) and data presented in Graphpad Prism. This pipeline was used to reanalyse data from GSE156553, GSE222505, E-MTAB-11784 and E-MTAB-11769 is presented in Fig. 4D,E.

### RT-qPCR

RNA was extracted using the Rneasy Plus Mini Kit (Qiagen, UK) according to the manufacturer's instructions. cDNA was prepared using the QuantiTect Reverse Transcription Kit as per manufacturer's instructions (Qiagen, UK). RT-qPCR was performed using the JumpStart Taq ReadyMix (Sigma-Aldrich), 50 ng cDNA and specific probes. Real-time PCR was performed with the CFX96-3 Real-Time PCR Detection System (Bio-Rad). Expression levels were normalized to the reference gene *HRPT*. Gene-specific primers are listed in Table S4.

### Tyrosine phosphoproteomics of tumour xenografts

Powdered tumour samples (3× WT and 3× PTPRK KO; Robertson and Leek, 2006) were solubilized in lysis buffer [8 M urea, 50 mM triethylammonium bicarbonate (TEAB), phosSTOP (Roche/Merck)], sonicated on ice (four cycles of 15 s on, 15 s off) and centrifuged at 16,000 ***g*** for 10 min. Cleared lysates were quantified using the Pierce BCA Protein Assay Kit following manufacturer's instructions. For each sample, an aliquot corresponding to 844.8 µg protein was reduced with 10 mM 1,4-dithiothreitol (DTT) for 45 min at room temperature and alkylated with 25 mM iodoacetamide (IAM) for 30 min at room temperature with shaking in the dark. Next, samples were pre-digested with LysC (0.05 AU; Wako) for 3 h at 30°C with shaking before diluting 5× with 50 mM TEAB and adding 8.5 µg trypsin (Promega) for overnight digestion at 37°C with shaking. Digested samples were acidified with 55 µl 10% trifluoroacetic acid (TFA) and centrifuged at 16,000 ***g*** for 5 min before the supernatant was desalted on 200 mg SepPak C18 cartridges (Waters). Each cartridge was washed twice with 1 ml 100% acetonitrile (ACN), then five times with 1 ml with 0.1% TFA. Next, the sample was loaded (3×) and the cartridge was washed ten times with 1 ml 0.1% TFA. Samples were eluted by adding 500 µl 50% ACN followed by 500 µl 70% ACN. Combined eluates were dried down in a vacuum centrifuge.

Dried peptide samples were resuspended in 100 µl 100 mM TEAB, then 41 µl of tandem mass tag (TMT) label (Thermo Fisher Scientific) in 100% ACN was added followed by a 2 h incubation at room temperature with shaking. WT replicates 1–3 were labelled with TMT126–128 and PTPRK KO replicates 1–3 with TMT 129–131. All samples were labelled with minimum 99% efficiency [evaluated by liquid chromatography–mass spectrometry (LC–MS) analysis] and were pooled in 1:1:1:1:1:1 ratio based on the total MS signal. The pooled sample was dried down in a vacuum centrifuge, then resuspended in 1 ml 0.1% TFA and desalted on a Chromabond HR-X cartridge (Macherey-Nagel). Briefly, the cartridge was washed with 5 ml 100% ACN, then equilibrated with 5 ml 0.1% TFA. Next, the sample was loaded (3×) and the cartridge was washed seven times with 1 ml 0.1% TFA. The sample was eluted by adding 500 µl 40% ACN, followed by 500 µl 70% ACN and 500 µl 70% ACN, 1% formic acid. 95% of the eluted sample was taken for phosphoproteomics, and the remaining 5% was saved for total proteome analysis.

First, tyrosine phosphorylated peptides were enriched using the PTMScan Pilot Phospho-Tyrosine Rabbit mAb (P-Tyr-1000) Kit (Cell Signaling Technology) following manufacturer's instructions. Dried sample was resuspended in 1× immunoaffinity purification (IAP) buffer and incubated with 1×80 µl immunoaffinity beads for 2.5 h at 4°C with gentle rotation. Beads were then washed 2×1 ml with IAP buffer and 3×1 ml with chilled LC–MS-grade water. To elute phosphopeptides, beads were resuspended in 55 µl 0.15% TFA, incubated with mixing for 10 min at room temperature, followed by centrifugation at 2000 ***g*** for 30 s. Eluate was transferred to fresh Protein LoBind tube (Eppendorf) and elution was repeated once more. Combined eluates were quantified with Pierce Quantitative Colorimetric Peptide Assay (Thermo Fisher Scientific), dried down and subjected to a second phosphopeptide enrichment step using TiO_2_ chromatography in batch mode (Thingholm and Larsen, 2016).

Dried samples were resuspended in 25 µl 0.1% TFA and further diluted with 350 µl loading buffer (1 M glycolic acid, 80% ACN, 5% TFA) before adding 0.6 mg TiO_2_ beads (GL Sciences) for 30 min incubation at room temperature with shaking. Beads were centrifuged at 1500 ***g*** for 10 s and supernatant incubated with 0.3 mg of fresh TiO_2_ beads for 20 min at room temperature with shaking. Next, TiO_2_ beads from the two incubations were combined and washed with 100 µl of each wash buffer 1 (80% ACN, 1% TFA) and wash buffer 2 (10% ACN, 0.1% TFA). Washed beads were dried down in vacuum centrifuge and incubated with 100 µl elution buffer (1.5% ammonia water, pH 11.3) for 20 min at room temperature with shaking. Eluted phosphopeptides were desalted on an in-house-made OLIGO R3 Reversed-Phase column with C18 plug (resin from Thermo Fisher Scientific). Briefly, the column was washed with 100 µl 100% ACN, then equilibrated four times with 100 µl 0.1% TFA. Sample was loaded onto the column (3×), before washing four times with 150 µl 0.1% TFA. Peptides were eluted with 75 µl 40% ACN, 0.1% TFA followed by two elutions with 65 µl of 70% ACN, 0.1% TFA. Combined eluates were dried down in a vacuum centrifuge and submitted for LC–MS analysis (performed at the CIMR proteomic facility).

Enriched tyrosine phosphopeptides were analysed on an Orbitrap Fusion Lumos Tribrid mass spectrometer coupled to a Dionex UHPLC system (Thermo Fisher Scientific). The sample was injected twice using complementary acquisition methods. For the MS3-SPS method, an MS1 scan in a range 380–1500 *m*/*z* was acquired in the Orbitrap with 120,000 resolution and 50 ms max injection time. For MS2, peptides were selected using 0.7 *m*/*z* isolation width with 120 ms max injection time, fragmented using collision induced dissociation (CID) with collision energy (CE) 34, and analysed in the IonTrap. Finally, the top ten fragment ions from MS2 were selected using 2 *m*/*z* isolation width with 150 ms max injection time, fragmented using higher-energy C-trap dissociation (HCD) with CE of 65, and analysed in the Orbitrap with 60,000 resolution. For the MS2 method, an MS1 scan in a range 350–1500 *m*/*z* was acquired in the Orbitrap with 120,000 resolution and 50 ms max injection time. For MS, peptides were selected using 1.6 *m*/*z* isolation width with 118 ms max injection time, fragmented using HCD with CE 35, and analysed in the Orbitrap with 60,000 resolution. Raw MS data from the two runs were searched against a human reference proteome and database of common contaminants with TMTsixplex on lysines and peptide N-termini and carbamidomethyl on cysteines as fixed modifications, and with oxidation on methionines and phosphorylation on serine/threonine/tyrosine as variable modifications using ProteomeDiscoverer software (Thermo Fisher Scientific). Abundance values were median normalized in each TMT channel, and only phosphopeptides with abundance values in all six TMT channels were taken forward. Significance analysis was carried out in Perseus (https://maxquant.net/perseus/) using *t*-test and permutation-based FDR to correct for multiple hypothesis testing.

## Supplementary Material



10.1242/joces.261914_sup1 Supplementary information

Table S1. Pathology report on middle aged Ptprk KO mice

Table S2. RNA Seq analysis of HT29 xenografts including pairwise differentially expressed genes (DEGs).

Table S3. Tyrosine phosphoproteomics analysis of HT29 xenografts.
