# Supplementary material for: The receptor protein tyrosine phosphatase PTPRK promotes intestinal repair and catalysis-independent tumour suppression
Source: J Cell Sci. 2024 Jul 22;137(14):jcs261914. doi: 10.1242/jcs.261914 (PMC11298714; doi:10.1242/jcs.261914)
Supplement: Supplementary information [file joces-137-261914-s1.pdf]

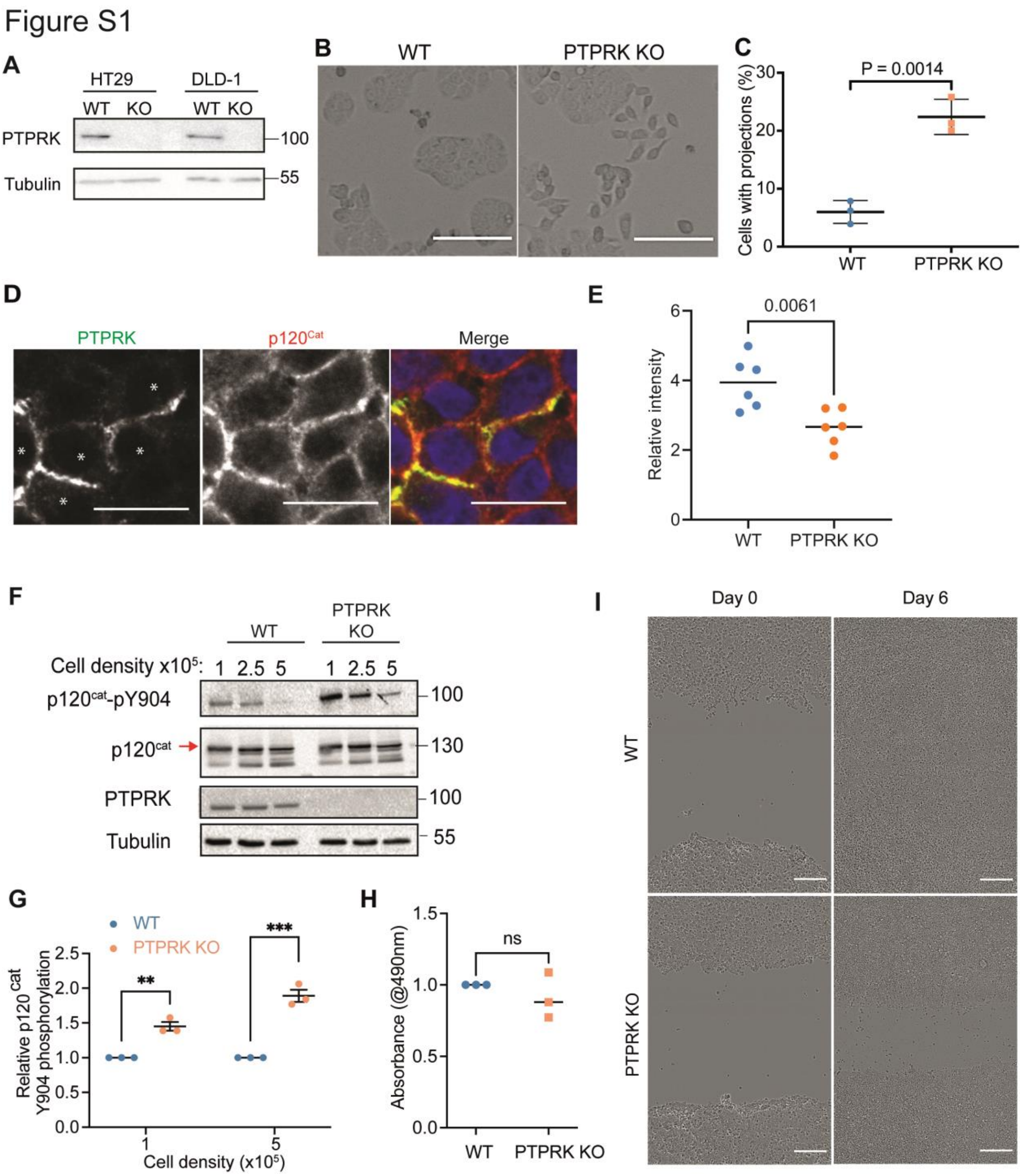

Fig. S1. Generation and characterisation of CRISPR/Cas9 PTPRK KO cell lines

A. Wildtype and PTPRK KO HT29 and DLD1 cells were cultured for 4 days and subjected to immunoblot analysis.

B. Representative phase contrast images of sub-confluent WT and PTPRK KO HT29 cells after 4 days in culture.

C. Quantification of cell projections from phase contrast images of WT and PTPRK KO (N=100; n=3). Error bars represent mean± s.d.. Unpaired two tailed, t test.

Journal of Cell Science • Supplementary information

D. $5x10^5$ WT and PTPRK KO HT29s were co-cultured for 3 days then fixed and stained with DAPI and antibodies against $p120^{Cat}$ and PTPRK. A representative confocal microscopy image is shown. White asterisks indicate WT (PTPRK positive) cells. Scale bar = 20 μm.

E. Quantification of relative $p120^{Cat}$ staining intensity. Average intensity of 5 cell-cell contacts per replicate image. N=5, n=6. Unpaired, two -tailed t test.

F. Wildtype or PTPRK KO HT29s were cultured for 4 days after plating at indicated cell density in a 12 well plate and subjected to immunoblot analysis with indicated antibodies.

G. Densitometric quantification of $p120^{Cat}$ phosphorylation normalized against total $p120^{Cat}$. Error bars denote mean ±s.e.m. (n = 3). Unpaired, two-tailed t test.

H. MTS proliferation assay absorbance at 490 nm for HT29 cell lines (n=3). Unpaired, two-tailed t test. ns = p> 0.05.

I. Representative phase contrast image of scratch wound with unstimulated wildtype and PTPRK KO HT29s on days 0 and 6.

Journal of Cell Science • Supplementary information

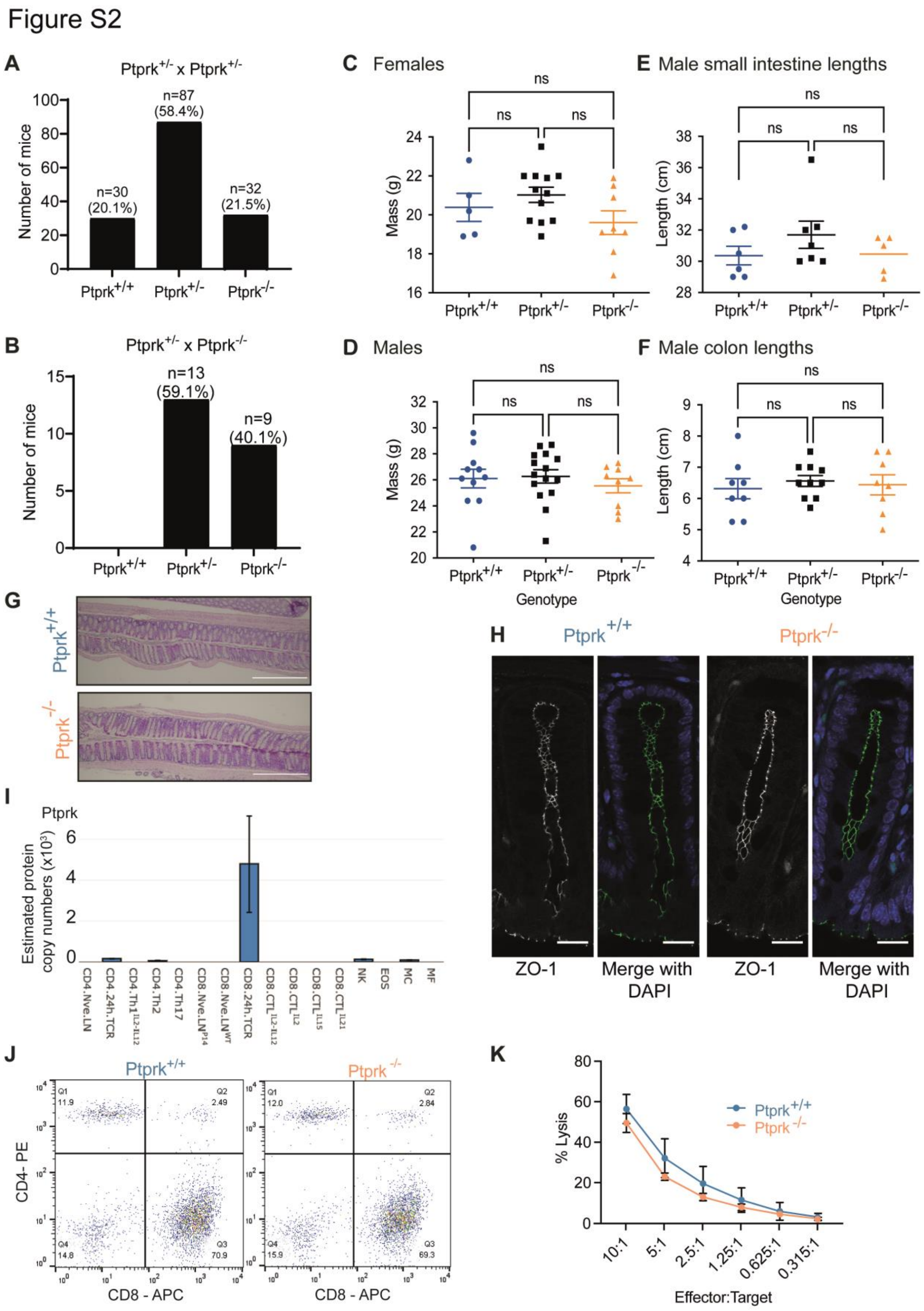

Fig. S2. Characterisation of *Ptprk* knockout mice

A. Quantification of weaned mice from 10 rounds of breeding *Ptprk*$^{+/-}$ x *Ptprk*$^{+/-}$ mice.

Journal of Cell Science • Supplementary information

B. Quantification of weaned mice from 3 rounds of breeding *Ptprk*$^{+/-}$x *Ptprk*$^{+/-}$ mice.

C and D. Weights of 6-week-old female (C) and male (D) *Ptprk*$^{+/+}$ (n= 5 and 11, respectively), *Ptprk*$^{+/-}$ (n=12 and n=15, respectively) and *Ptprk*$^{-/-}$ mice (n=8 and n=9, respectively). Error bars denote mean±s.e.m.. Unpaired, two-tailed t tests. ns = p >0.05.

D and E. Intestine (D) and colon (E) length measurements of 6-week-old male *Ptprk*$^{+/+}$ (n=6 and n=8, respectively), *Ptprk*$^{+/-}$ (n=7 and n=10, respectively) *Ptprk*$^{-/-}$ (n=5 and n=8, respectively) mice. Error bars denote mean ±s.e.m.. Unpaired, two-tailed t tests. ns = p >0.05.

G. Representative hematoxylin and eosin stained sections of distal colon taken from 7 week old *Ptprk*$^{+/+}$ and *Ptprk*$^{-/-}$ mice. Scale bar= 1 mm.

H. Immunofluorescence of ZO-1 in tight junctions between colonic crypts, colon sections taken from 7-week-old *Ptprk*$^{+/+}$ and *Ptprk*$^{-/-}$ mice. DAPI and ZO-1 channels have been overlaid in the merged images.

I. Estimated protein copy numbers for PTPRK in mouse immune cells. Generated using ImmPRes (Brenes et al., 2023).

J. Representative example of flow cytometric analysis of CD4+ and CD8+ T cells from *Ptprk*$^{+/+}$ and *Ptprk*$^{-/-}$ mice.

K. Cytotoxicity assay with CD8+ cytotoxic T lymphocytes from *Ptprk*$^{+/+}$ and *Ptprk*$^{-/-}$ mice (n=3). Error bars denote mean ±s.e.m. Unpaired, two-tailed t test. All comparisons gave p >0.05.

Journal of Cell Science • Supplementary information

Figure S3

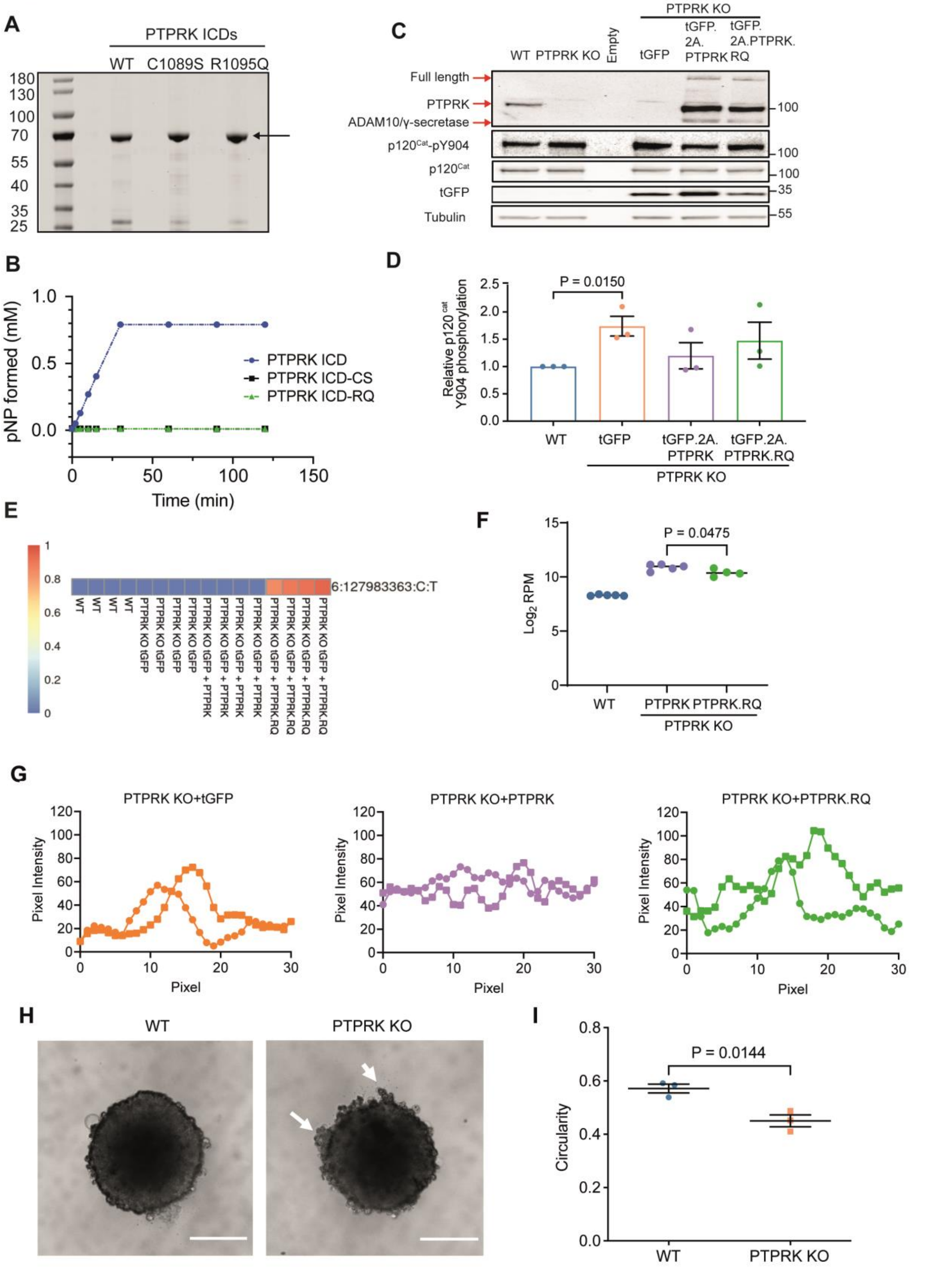

Fig. S3. Characterisation of PTPRK mutants and doxycycline-inducible cell lines A. Ni-NTA and size exclusion chromatography purified recombinant proteins were resolved by SDS-PAGE followed by Coomassie staining.

Journal of Cell Science • Supplementary information

B. The phosphatase activity of 2 μg recombinant proteins was assayed using 20 mM pNPP and quantified at 405 nm at indicated time points.

C. Wildtype or PTPRK KO HT29s, with stably-integrated doxycycline-inducible tGFP, PTPRK or PTPRK-R1095Q, were cultured for 24 hours with 1 μg/ml doxycycline then lysed and subjected to immunoblot analysis.

D. Densitometric quantification of $p120^{Cat}$ phosphorylation normalized against total $p120^{Cat}$. Error bars denote mean ±s.e.m (n = 3). Unpaired, two tailed t test. P values shown for comparisons with $p \leq 0.05$. All other comparisons $p > 0.05$.

E. RNA-seq reads corresponding to PTPRK codon 1095 from PTPRK KO tumours expressing dox-inducible PTPRK or PTPRK.R1095Q.

F. Log_2 (RPM) from RNA sequencing for *PTPRK* in indicated samples. Unpaired, two tailed t test, n=4 or 5 as indicated by individual data points.

G. Fluorescence intensity across junctions between tGFP-expressing cells from xenografts stained with p120Cat-pY904.

H. Wildtype and PTPRK KO HT29 cells were cultured as spheres on 1.5% agarose. White arrows indicate projections into agarose.

I. Quantification of circularity of spheres using imageJ analysis (N=15; n=3). Error bars denote mean ±s.e.m. Unpaired, two tailed t test.

Journal of Cell Science • Supplementary information

Figure S4

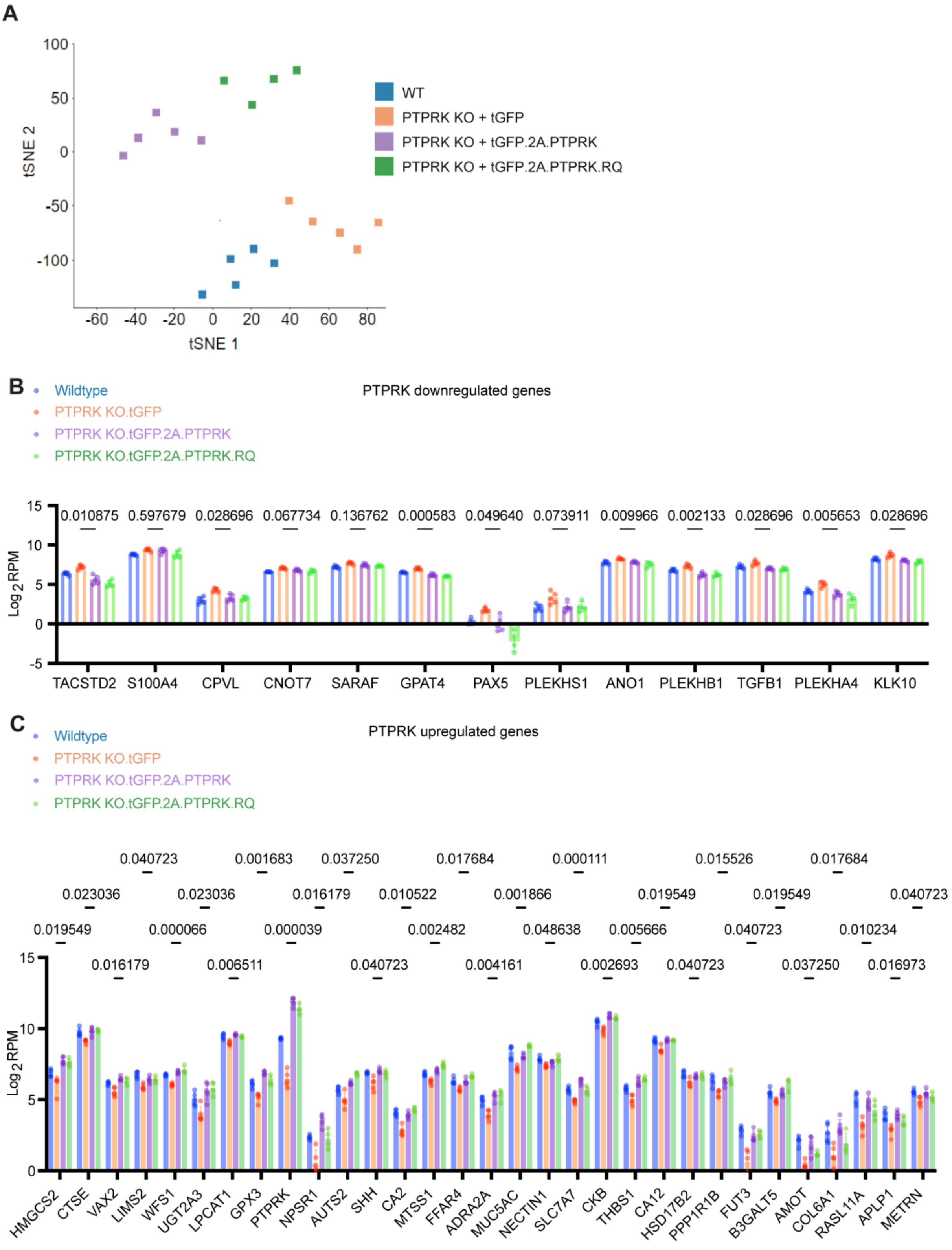

Fig. S4. RNA-seq analysis of HT29 xenografts

A. t-SNE plot of RNA-seq data for four or five xenografts per cell line.

Journal of Cell Science • Supplementary information

B. Log_2 (RPM) for differentially PTPRK downregulated genes derived from RNASeq analysis. P values for PTPRK KO.tGFP vs PTPRK KO.tGFP+PTPRK from multiple unpaired, two tailed t tests are shown.

C. Log_2 (RPM) for differentially PTPRK upregulated genes derived from RNASeq analysis. P values for PTPRK KO.tGFP vs PTPRK KO.tGFP+PTPRK from multiple unpaired, two tailed t tests are shown.

Journal of Cell Science • Supplementary information

Figure S5

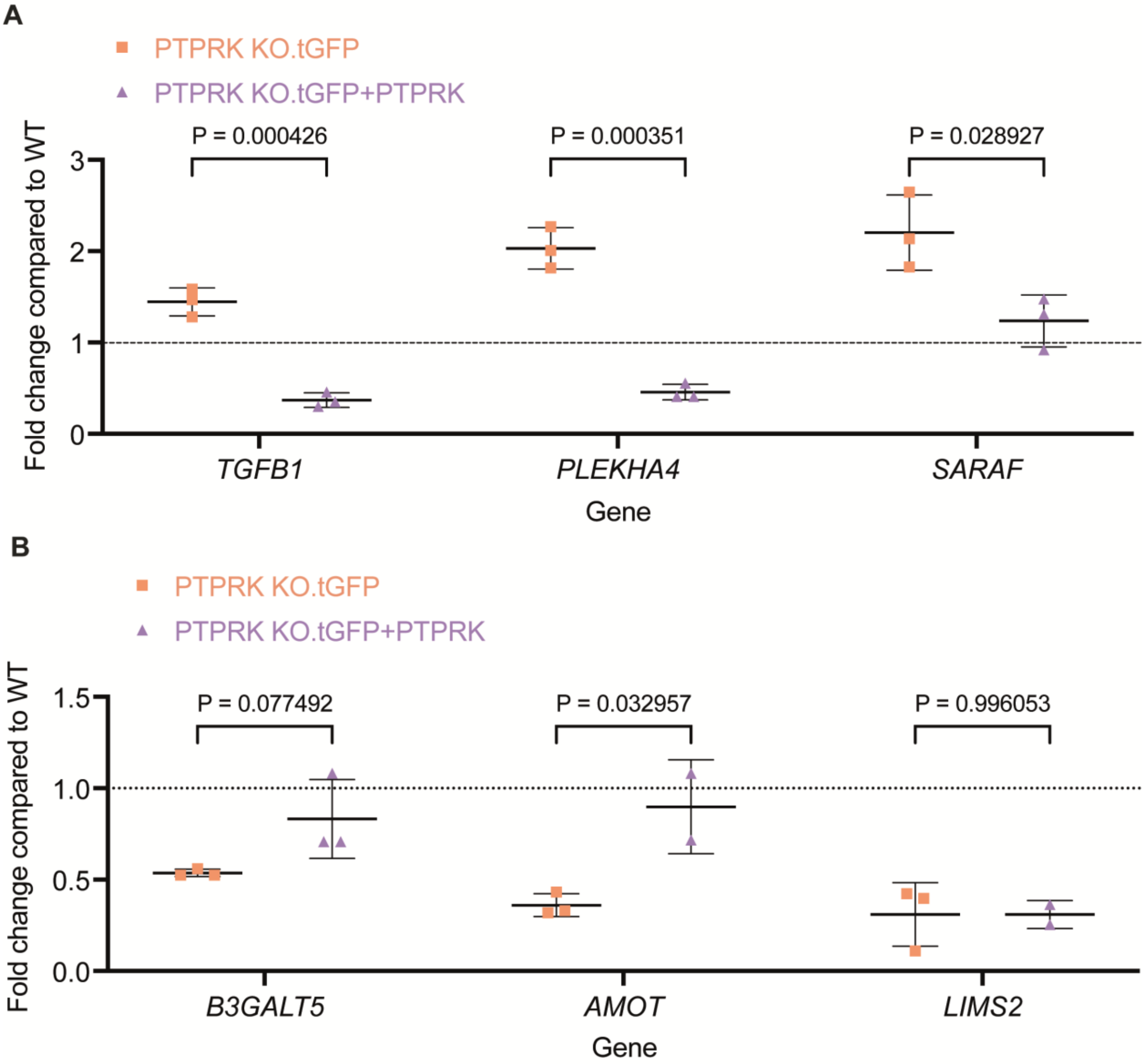

Fig. S5. qPCR validation of differentially expressed genes in 2D cell culture

A and B. qPCR analysis of cDNA reverse transcribed from RNA isolated from confluent HT29s using the indicated probes. Dotted line indicates WT levels which values are normalized against. Ct values relative to the housekeeping gene *HPRT* were normalized using $2^{-\Delta Ct}$. The means of technical duplicates are shown. Error bars denote mean ±s.e.m. Unpaired, two-tailed t test carried out for WT vs. PTPRK KO.tGFP and PTPRK KO.tGFP vs PTPRK KO.tGFP+PTPRK (n=3).

Journal of Cell Science • Supplementary information

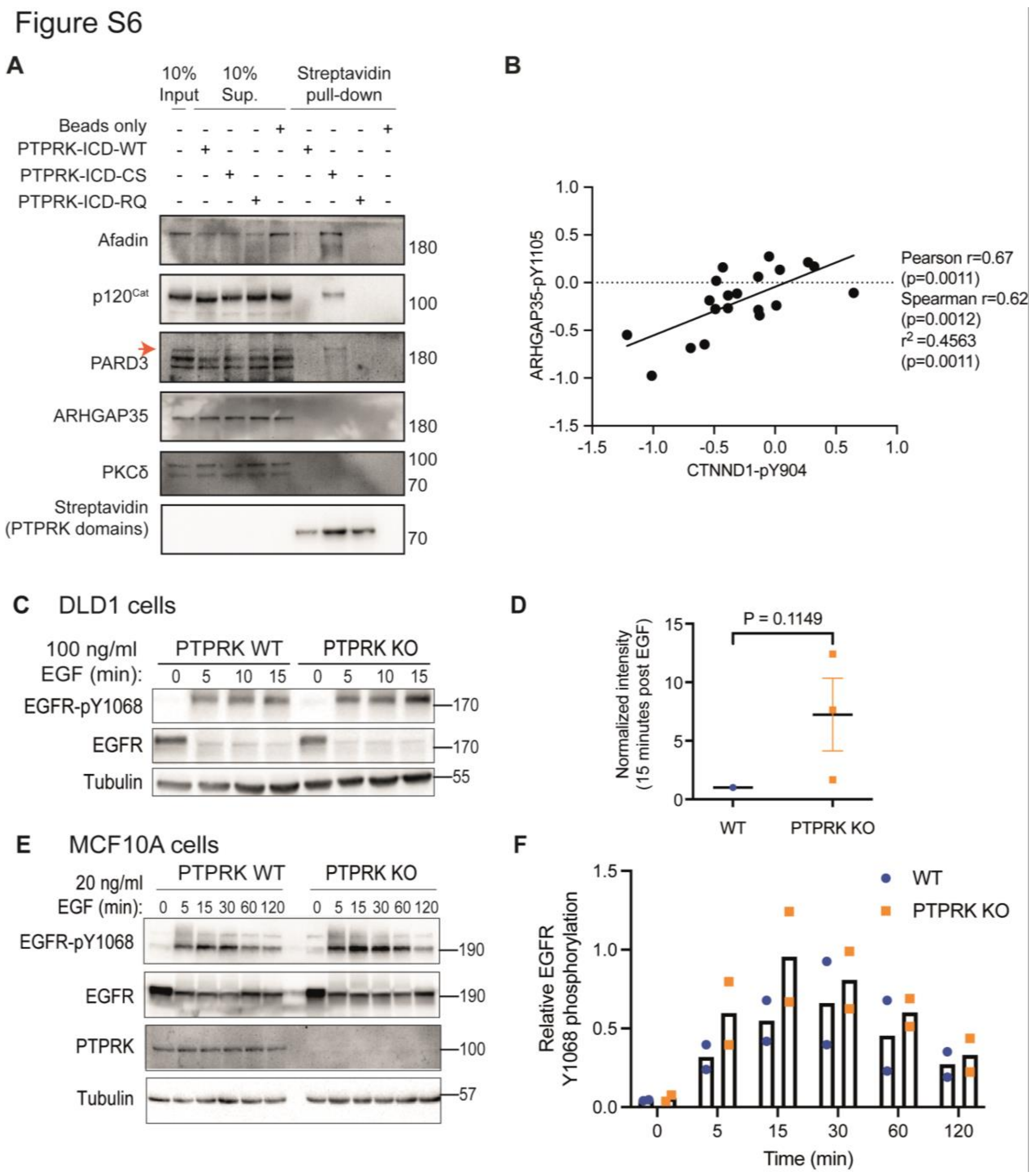

Fig. S6. PTPRK interactions and regulation of EGFR

A. Confluent, pervanadate-treated MCF10A lysates were used for recombinant protein streptavidin pull downs with indicated PTPRK intracellular domains, followed by immunoblotting with indicated antibodies. Catalytic mutants: CS = C1089S. RQ = R1095Q.

B. Phosphoprotein site level expression in colorectal cancer (mass spectrometry by CPTAC) derived from cBioportal. Simple linear regression, and parametric and nonparametric correlation analyses.

C. Wildtype or PTPRK KO DLD1s were cultured for 4 days, starved overnight, and treated as indicated, followed by lysis and immunoblot analysis with indicated antibodies.

Journal of Cell Science • Supplementary information

D. Densitometric quantification of EGFR Y1068 phosphorylation after 15 minutes of EGF stimulation, normalized against total EGFR (n=3). Error bars denote mean ±s.e.m. Unpaired, two-tailed t test.

E. Wildtype or PTPRK KO MCF10As were cultured for 4 days, starved overnight, and treated as indicated, followed by lysis and immunoblot analysis with indicated antibodies.

F. Densitometric quantification of EGFR Y1068 phosphorylation normalized against total EGFR (n=2).

Figure S7.

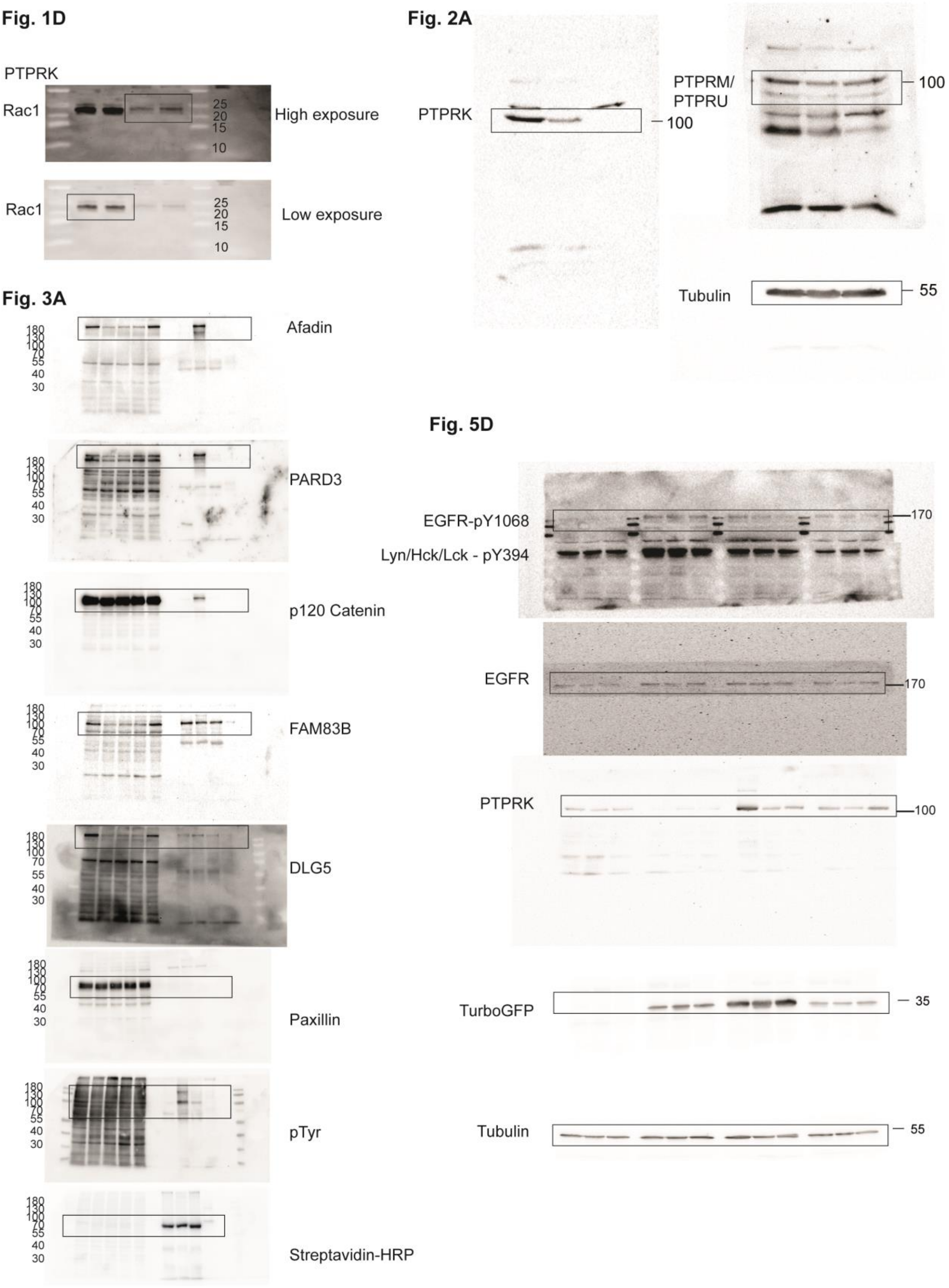

Journal of Cell Science • Supplementary information

Fig. 5F

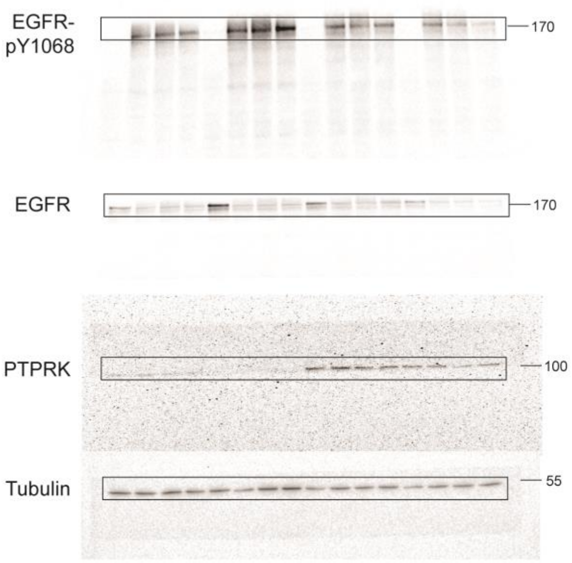

Fig. 5H

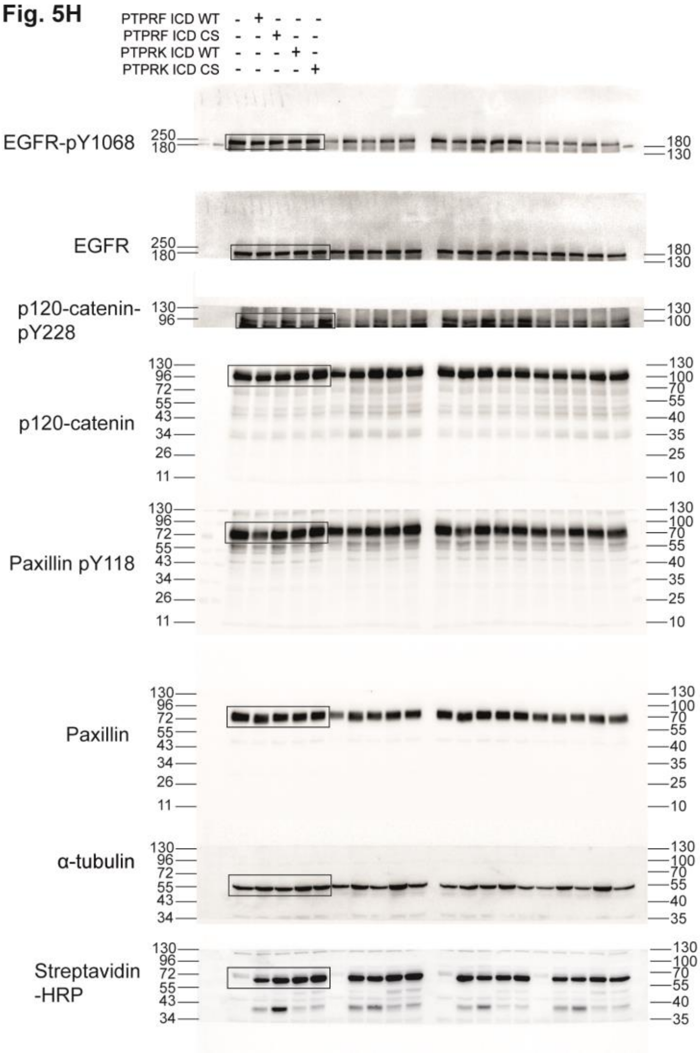

Fig. S1A

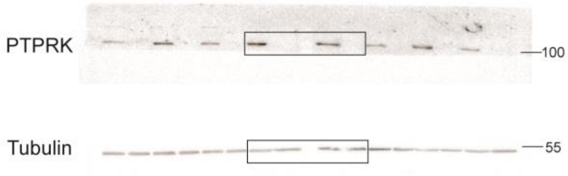

Fig. S1F

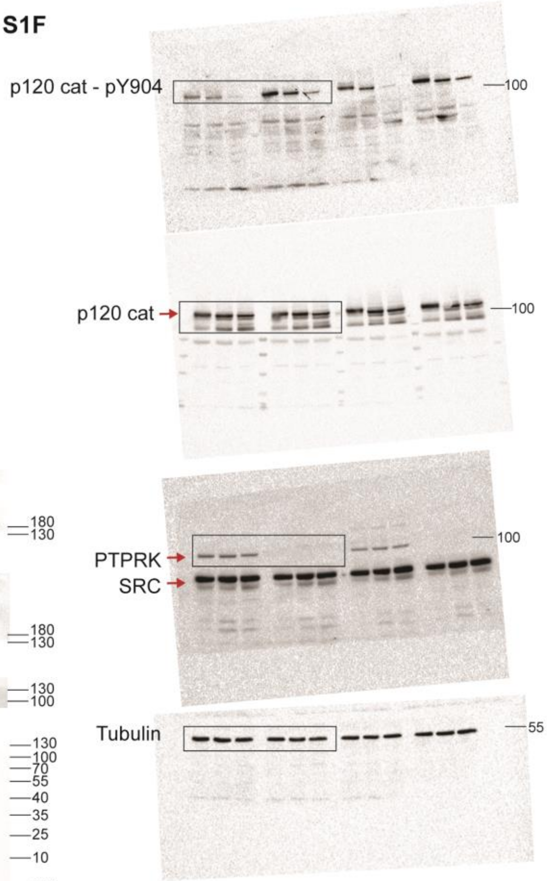

Fig. S3C

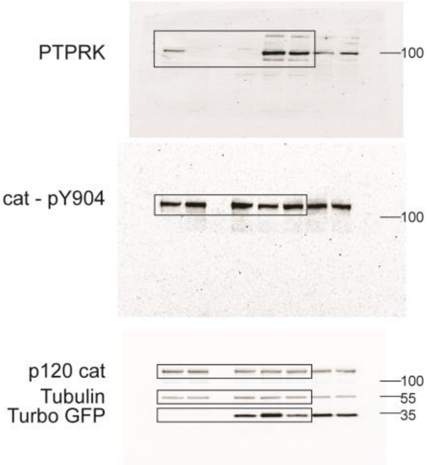

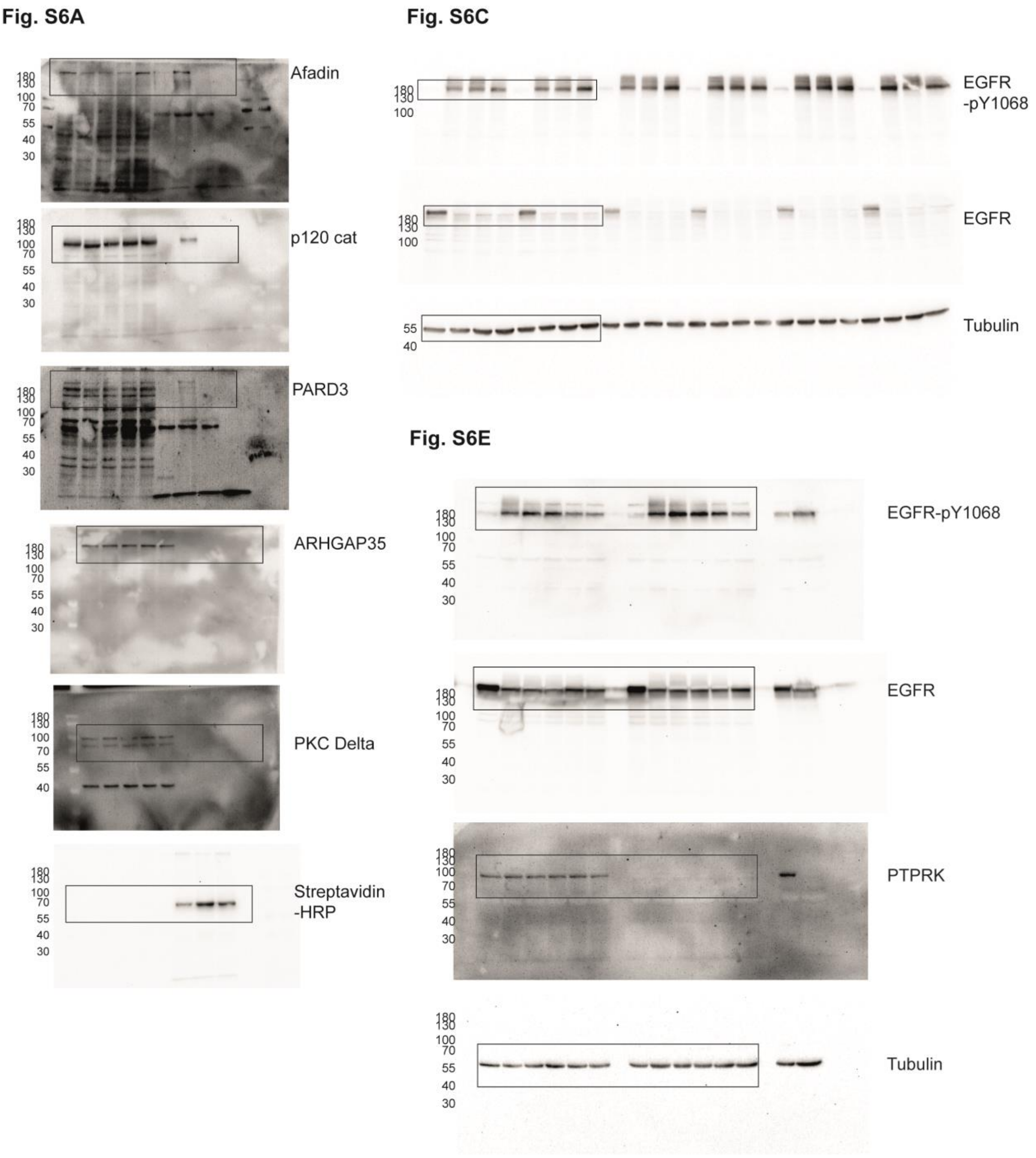

Fig. S7. Western blot transparency

Table S1. Pathology report on middle aged Ptprk KO mice

Available for download at
https://journals.biologists.com/jcs/article-lookup/doi/10.1242/jcs.261914#supplementary-data

Table S2. RNA Seq analysis of HT29 xenografts including pairwise differentially expressed genes (DEGs).

Available for download at
https://journals.biologists.com/jcs/article-lookup/doi/10.1242/jcs.261914#supplementary-data

Table S3. Tyrosine phosphoproteomics analysis of HT29 xenografts.

Available for download at
https://journals.biologists.com/jcs/article-lookup/doi/10.1242/jcs.261914#supplementary-data

Table S4. Experimental models

**Reagent**	**Reference or source**	**Catalog number or other identifier**	**Species**
Ptprk$^{-/-}$ C57BL/6N (*M. musculus*)	This study	Ptprk$^{-/-}$	Mouse
NU(NCr)-Foxn1nu (*M. musculus*)	Charles River	088 (Homozygous)	Mouse
HT29 cells	ATCC	HTB-38	Human
HT29 PTPRK KO	This study		Human
HT29 PTPRK KO + stable tGFP	This study		
HT29 PTPRK KO + stable tGFP.2A.PTPRK	This study		Human
HT29 PTPRK KO + stable tGFP.2A.PTPRK-R1095Q	This study		Human
MCF10A	ATCC	CRL-10317	Human
MCF10A PTPRK KO	Fearnley et al, 2019		Human
HEK293T	Fearnley et al, 2019		Human
DLD1 cells	Gift from the Cook lab, Babraham Institute		Human
DLD1 PTPRK KO	This study		

Antibodies

**Target**	**Species/mono- or polyclonal**	**Company/source**	**Catalog number**	**Use and dilution**
**Primaries**				
Tubulin, Alpha	Mouse monoclonal	Sigma	T6199	Western Blot 1:5000
Y904 p120 Catenin	Rabbit polyclonal	Cell signaling technologies	2910	Western Blot 1:1000
Afadin	Mouse monoclonal	BD Transduction Labs	610732	Western Blot 1:1000
PTPRK	Humanised monoclonal	Fearnley et al. 2019	NA	Western Blot 1:1000

Journal of Cell Science • Supplementary information

PTPRK	Rabbit monoclonal	Fearnley et al. 2019	N/A	Immunofluorescence 1:1000
PARD3	Rabbit polyclonal	Atlas	HPA030443	Western Blot 1:1000
EGFR	Rabbit monoclonal	Cell signaling technologies	4267	Western Blot 1:1000
EGFR pY1068	Rabbit monoclonal	Cell signaling technologies	3777	Western Blot 1:1000
Gamma tubulin	Mouse monoclonal	ThermoFisher Scientific	MA1-19421 (lot#74685130)	Immunofluorescence 1:1000
Paxillin	Rabbit monoclonal	Cell signal technologies	12065	Western Blot 1:1000
DLG5	Mouse monoclonal	Santa Cruz Biotechnology	SC374594	Western Blot 1:1000
p120 Catenin	Mouse monoclonal	BD Transduction Laboratories	610133	Western Blot 1:1000 Immunofluorescence 1:400
pMLC2	Mouse monoclonal	Cell Signaling Technology	3675S	Immunofluorescence 1:400
ZO-1	Mouse monoclonal	ThermoFisher Scientific	Z01-1A12	Immunofluorescence 1:400
Rac1	Mouse monoclonal	Millipore	05-389	Western Blot 1:1000
PTPRM	Mouse monoclonal	Santa Cruz	Sc-56959	Western Blot 1:500
Fam83B	Rabbit polyclonal	Atlas Antibodies	HPA031464	Western Blot 1:1000
Phosphotyrosine	Rabbit monoclonal	Cell Signaling Technology	8954S	Western Blot 1:1000
Mouse CD4	PE-conjugated Rat monoclonal	Biolegend	100408	Flow cytometry 1:100
Mouse CD8a	APC-conjugated rat monoclonal	Biolegend	100712	Flow cytometry 1:100
**Secondaries**				
Atto-488 Anti-mouse IgG		Sigma	62197	Immunofluorescence 1:400
568?				

Journal of Cell Science • Supplementary information

Rabbit IgG-HRP conjugated		Jackson ImmunoResearch / Stratech	711-035-152	Western Blot 1:3333
Mouse IgG-HRP conjugated		Jackson ImmunoResearch / Stratech	715-035-150	Western Blot 1:3333
**Other affinity reagents**				
Streptavidin-HRP conjugated		Invitrogen	434323	Western Blot 1:15000
Biotin-SP-conjugated Affinipure goat anti-species IgG		Invitrogen	31820	Flow cytometry 1:100
R-Phycoerythrin Streptavidin		Jackson ImmunoResearch	016-110-084	Flow cytometry 1:50
Phalloidin 558/568		ThermoFisher	B3475	Immunofluorescence 1:400

Recombinant DNA

**Reagent**	**Reference or source**	**Catalog number or other identifier**	**Species**
pCW57-tGFP-2A-MCS	Addgene	Cat #71783	NA
pCW57_tGFP_P2A_PTPRK	Fearnley et al, 2019		Human
pCW57_tGFP_P2A_PTPRK_R1095Q	This study		Human
pGEX-PAK-CRIB	A gift from Welch lab, Babraham Institute		NA
pSp.Cas9-2A-eGFP-PTPRK.gRNA	Fearnley et al, 2019		Human

Journal of Cell Science • Supplementary information

His.TEV.Avi.PTPRK.ICD	Fearnley et al, 2019		Human
His.TEV.Avi.PTPRK.ICD.C1089S	Fearnley et al, 2019		Human
His.TEV.Avi.PTPRK.ICD.R1095Q	This study		Human

Primers

**Purpose**	**Name**	**Sequence (5'->3')**
Mouse genotyping	PTPRK_Forward_Genotyping<br>PTPRK_Reverse_Genotyping	CAAAGCTGCTTGAAACTTCT<br>AAGACTGTGGACAGACAC
RNAseq qPCR validation	AMOT F<br>AMOT R	GTTGGAGCTGCCTTCTATGT<br>CCCTCATCTTGGTGCATCTT
	SARAF F<br>SARAF R	CCTCGGCTTGCATTTGTTTC<br>AGGTGGTATAGCGGTCATAGT
	LIMS2 F<br>LIMS2 R	TCTGTGCCAAGTGTGAGAAG<br>CGAAGAGCTGGTTGTAGTGAG
	B3GALT5 F<br>B3GALT5 R	CCAAGAGCGTCCCATACATTA<br>CTGAAGAGGCATACGGAGAAG
	PLEKHA4 F<br>PLEKHA4 R	ACCAAAGCTTGGAGACAGATAC<br>CTGCTTCTAGTTGCTCCTTCTC
	TGFB1 F<br>TGFB1 R	CGTGGAGCTGTACCAGAAATAC<br>CACAACTCCGGTGACATCAA
	HPRT F<br>HPRT R	CGAGATGTGATGAAGGAGATGG<br>TTGATGTAATCCAGCAGGTCAG
Mutagenesis	PTPRK R1095Q F<br>PTPRK R1095Q R	GTGCTGGACAAACTGGCTGCTACATTGTGATTGACATC<br>GTAGCAGCCAGTTTGTCCAGCACCAGCACTGCAATG

Journal of Cell Science • Supplementary information

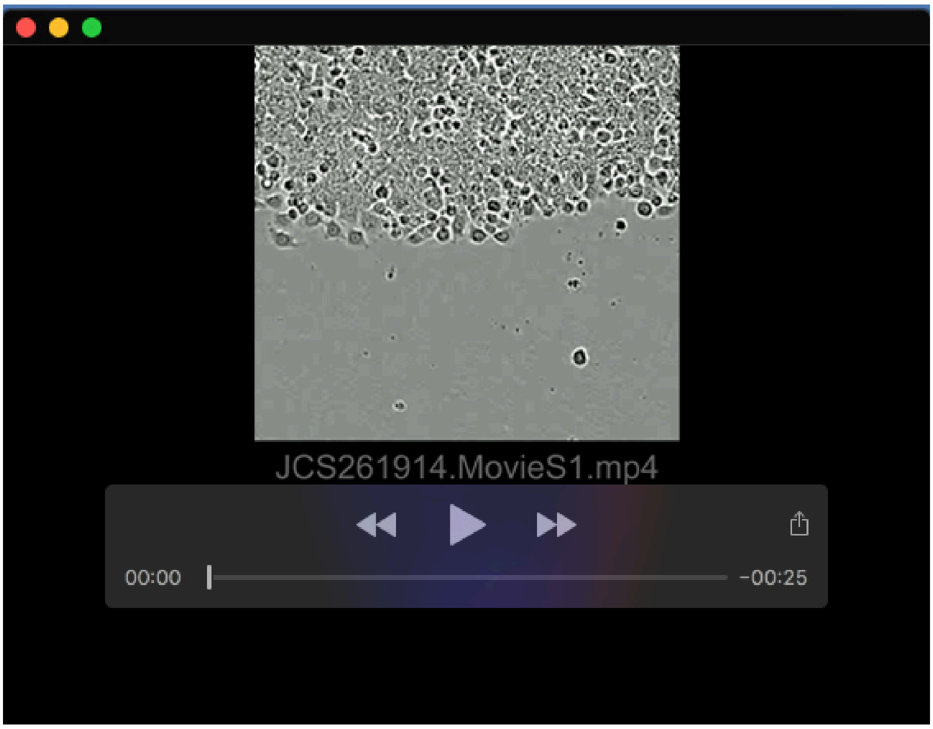

Movie 1. PTPRK WT Scratch wound

Video of leading edge of scratch wound over the initial 32 hours post scratch. Captured using Incucyte® software

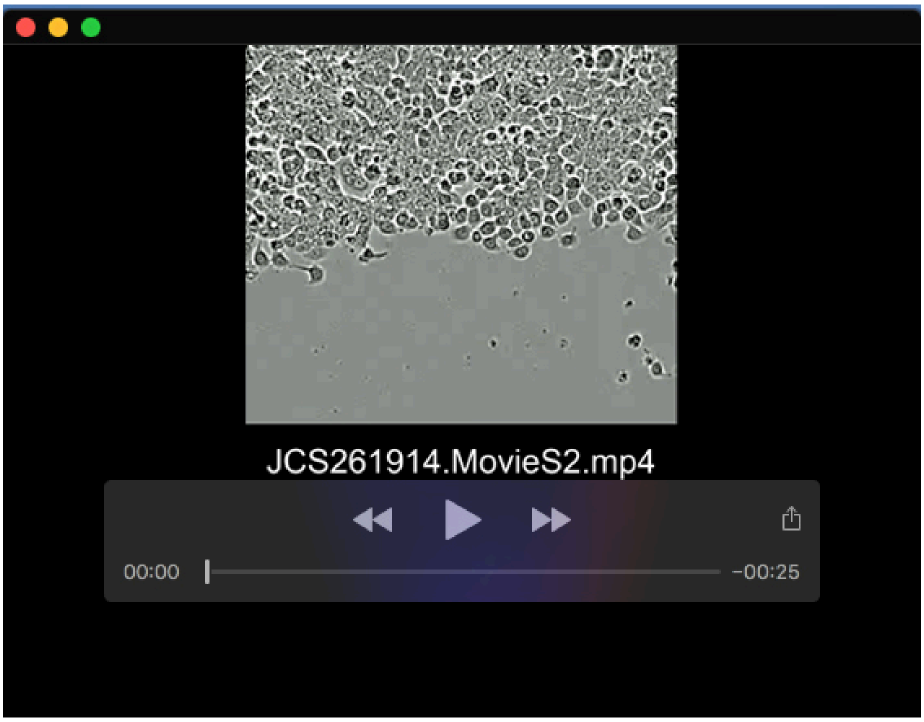

Movie 2. PTPRK KO scratch wound

Video of leading edge of scratch wound over the initial 32 hours post scratch. Captured using Incucyte® software

Journal of Cell Science • Supplementary information
