# Supplementary material for: The receptor protein tyrosine phosphatase PTPRK promotes intestinal repair and catalysis-independent tumour suppression
Source: J Cell Sci. 2024 Jul 22;137(14):jcs261914. doi: 10.1242/jcs.261914 (PMC11298714; doi:10.1242/jcs.261914)
Supplement: Supplementary file 1 [file joces-137-261914_review_history.pdf]

# The receptor protein tyrosine phosphatase PTPRK promotes intestinal repair and catalysis-independent tumour suppression

Katherine A. Young, Katarzyna Wojdyla, Tiffany Lai, Katie E. Mulholland, Silvia Aldaz Casanova, Robin Antrobus, Simon R. Andrews, Laura Biggins, Betania Mahler-Araujo, Philippa R. Barton, Keith Anderson, Gareth W. Fearnley and Hayley J. Sharpe
DOI: 10.1242/jcs.261914

**Editor**: Andrew Ewald

**Review timeline**

| | |
|---|---|
| Original submission: | 18 December 2023 |
| Editorial decision: | 6 February 2024 |
| First revision received: | 16 May 2024 |
| Accepted: | 28 May 2024 |


---

**Original submission**

First decision letter

MS ID#: JOCES/2023/261914

MS TITLE: The receptor protein tyrosine phosphatase PTPRK promotes intestinal repair and catalysis-independent tumour suppression

AUTHORS: Katherine A Young, Katie E Mulholland, Laura Biggins, Robin Antrobus, Gareth W Fearnley, Philippa Barton, Tiffany Lai, Keith Anderson, Katarzyna Wojdyla, Simon R Andrews, Betania Mahler-Araujo, and Hayley J Sharpe

We have now reached a decision on the above manuscript.

To see the reviewers' reports and a copy of this decision letter, please go to: https://submit-jcs.biologists.org and click on the 'Manuscripts with Decisions' queue in the Author Area. (Corresponding author only has access to reviews.)

As you will see, both reviewers share enthusiasm for the study and its conclusions. They also make a number of focused suggestions for how to improve the manuscript. They suggest that a revised version might prove acceptable, if you can address their concerns. If you think that you can deal satisfactorily with the criticisms on revision, I would be pleased to see a revised manuscript. We would then return it to the reviewers.

Please ensure that you clearly highlight all changes made in the revised manuscript. Please avoid using 'Tracked changes' in Word files as these are lost in PDF conversion.

I should be grateful if you would also provide a point-by-point response detailing how you have dealt with the points raised by the reviewers in the 'Response to Reviewers' box. Please attend to all of the reviewers' comments. If you do not agree with any of their criticisms or suggestions please explain clearly why this is so.

Reviewer 1

© 2024. Published by The Company of Biologists under the terms of the Creative Commons Attribution License (https://creativecommons.org/licenses/by/4.0/).

*Advance summary and potential significance to field*

Description of function of receptor tyr phosphatase, PTPRK in multiple intestinal models, particularly colon cancer lines as well as a mouse model showing this phosphatase is involved in recovery from a destructive/inflammation colitis model (DSS), and acts as a tumor suppressor in mouse colon and in in vivo mouse colon implantation models, that also involves limiting invasiveness of model colon cancer. Most importantly is demonstration of effects of this phosphatase which do not appear to involves changes in substrate phosphorylation particularly effects involving EGF and EMT. Notably what the alternative mechanisms are remains to be determined.

*Comments for the author*

Interesting study of a receptor protein tyr phosphatase PTPRK showing that is has what appears to several function that do not involve changing phosphorylation. This study of the many functions, appears to be somewhat confirmatory and the identification of functions that do not involve changing phosphorylation and its involvement in repair of damage/inflammation (DSS colitis) are the major advances. There are multiple technical issues but not major flaws if the major observations are felt to be sufficiently rigorously demonstrated in the manuscript. I was surprised by the brevity of the legends with my terms not explained with the assumption that the readership is well versed in study of mechanisms of cancer including colon cancer. I suggest that this assumption be damped down and more description of the cancer players be defined . Specific comments:

1) No statement in methods of which statistical forms of evaluation are applied. Please add to each legend, number of repetitions of experiments and method used for statistical comparison. Sometimes provided and sometimes not.
2) S IF hyper phos of P120 : what is shown in the statistical comparison of multiple experiments, is not what shown in single experiment in high density which in fact seems to show reduced phosphorylation
3) Fig 2 : define Ptprm and PTPru in legend; present numbers for surviving number of wt, het and homozygous ko to show distribution
4) Video 1 and 2: state which is which
5) S 2D: what is being merged.
6) Fig 2G/H: statistical comparison of invasion of wt/KO should be provided.
7) Fig 3A not clear what is being shown as WT and RQ seem similar at the magnification I can achieve; S F 3B I can not see any data for CS
8) Statement referred to Fig 3C (meant S 3C) that RQ expressed to lower amount than WT needs support by statistical evaluation of multiple repeated experiments. Fig 3C. This identification of effects of PTPRK that do not involve phosphatase activity is the most innovative aspect of the study and needs more in depth study that can be statistically evaluated to support what is shown in Fig 3E,F.
9) Fig 3D and E as labeled represent 3E and F. Also in text, these studies are mislabeled as Fig 2 D,E
10) Fig S5, there are p values shown but in addition the legend uses * to indicate different p values.
11) Fig S6B no data stated in text on ARHGAP5 are shown.

<u>Reviewer 2</u>

*Advance summary and potential significance to field*

Contrary to oncogenic kinases, the role of phosphatases is less well understood and remains largely unknow. In this manuscript the Sharpe laboratory have investigated PTPRK receptor tyrosine phosphatase in colon homeostasis and colon cancer. They report several PTPRK-supported tumor-suppressive mechanism including inhibition of EMT, a supportive role in cell-cell adhesion and inhibition of invasion and disruption of cell front-rear polarity. Excitingly they find that the tumro suppressive activity is independent of PTPRK kinase activity indicative of a non-canonical regulatory function for PTPRK.

© 2024. Published by The Company of Biologists under the terms of the Creative Commons Attribution License (https://creativecommons.org/licenses/by/4.0/).

Overall, this is a carefully prepared and for the most part clearly written manuscript. The data are well controlled, the experiments are of high-quality and range from in vitro biochemistry to in vivo tumor biology and interrogation of tissue homeostasis. The observation of PRPRK acting as a tumor suppressor independently of its phosphatase activity is interesting and will open new doors in the field. Finally, the observation that phospho-EGFR is not a direct substrate for the phosphatase is important.

*Comments for the author*

Major points:
Fig 3. The interpretation of the blot in Figure 3A is not entirely clear. In the text the authors indicate that these data demonstrate R1095Q mutant as phosphatase dead but not substrate trapping. Please explain why are FAM83B and DLG5 included and why do they associate with R1095Q. I suppose paxillin is included as negative control ? whereas the others are established substrates.
Please clarify this in the text.
Did reconstitution with R1095Q influence HT29 cell proliferation in vitro?
Fig 3D. Would it be possible to show the effect of R1095Q expression on junctional p-p120 intensity similarly to the wt?
Figure4. The authors cite many times Wang et al., 2005 showing that PTPRK suppresses TGFB1. However, looking at the RNAseq data there does not seem to be a significant effect in TGFB1 expression between ctrl and KO cells and TGFB is suppressed only in the reconstituted cell lines overexpressing wt or RQ PTPRK.
Would be good if the authors could please comment on these data linked to the published data they discuss in 4D.
The data indicating that EGFR phosphorylation is not regulated by PTPRK are interesting and seem convincing and are in line with the increased EGFR-P upon acute stimulation S6E. As the story started with collective migration and invasion, it would be nice if the authors could test PTPRKwt vs. RQ cells for their responses to EGF-induced cell migration and/or invasion.
Minor 1F. Perhaps consider rewording “correctly polarized g-tubulin” with for example … check methods. IF figure legends mentions staining of GM130 but this seems to be missing from the figure ?? or are the pictures labelled PTPRK the GM130 staining?

---

**First revision**

<u>Author response to reviewers' comments</u>

Rebuttal for “The receptor protein tyrosine phosphatase PTPRK promotes intestinal repair and catalysis-independent tumour suppression”

We would like to thank the reviewers for their time in providing feedback on our manuscript and to the editor for the opportunity to improve it. We have now attempted to address the concerns raised. This includes increasing the level of detail contained within figure legends as well as the addition of extra experiments and increasing the clarity of the text. All changes are marked up in yellow highlighter. The most significant insight is from the suggested wound healing assay. This shows that EGF stimulates wound closure, which is enhanced in the absence of PTPRK, and suppressed in the presence of either PTPRK or the catalytically inactive mutant. Under these conditions, it once again appears that catalysis is not critical in repressing the EGF response and adds to this interesting aspect of the manuscript. Below is a point-by-point response to comments.

**Reviewer 1.**
1) No statement in methods of which statistical forms of evaluation are applied.
Please add to each legend, number of repetitions of experiments and method used for statistical comparison. Sometimes provided and sometimes not.

We have now ensured that the replicates and details of statistical tests are included in each

© 2024. Published by The Company of Biologists under the terms of the Creative Commons Attribution License (https://creativecommons.org/licenses/by/4.0/). 

legend. We have tried to be as transparent as possible by plotting data points (rather than summary bars) where appropriate.

2) S IF hyper phos of P120 : what is shown in the statistical comparison of multiple experiments, is not what shown in single experiment in high density which in fact seems to show reduced phosphorylation

We interpreted this comment to be related to Figure S1F. We have performed wildtype vs knockout experiments at the two densities numerous times. We consistently see higher phosphorylation of p120 catenin in the KO cells under equivalent conditions. We have now included another blot to show this even more clearly (new Fig S1F). We hope that it is now clearer that P120 catenin phosphorylation decreases with increasing cell density and that the absence of PTPRK is associated with higher levels of phosphorylation at each density. In addition, we have replaced figures S1D and S1E with a co-culture experiment of PTPRK WT and KO HT29 cells stained with p120catenin and PTPRK. This shows that the fluorescence intensity of p120 catenin is lower at cell-cell contacts between PTPRK KO cells than between PTPRK WT cells. We had originally stained for E cadherin, however, in HT29 cells E cadherin is present in puncta that made the quantification of fluorescence intensity challenging.

3) Fig 2 : define Ptprm and PTPru in legend; present numbers for surviving number of wt, het and homozygous ko to show distribution

We have now clarified that Ptprm and Ptpru are paralogs of Ptprk in both the text (page 5) and legend of Figure 2. For the second point, we interpreted this as providing evidence for no effect on fertility. We have now included graphs in Figure S2 (A and B) outlining the outcomes of 10 rounds of het x het crosses, as well as 3 rounds of het x hom crosses, where we observed close to expected mendelian ratios. This is now stated more explicitly in the main text. The numbers of animals used for experiments is stated in the relevant figure legends (Figures 2C and 2F).

4) Video 1 and 2: state which is which

We have highlighted which video is which in the main text in addition to the information already in the supplement.

5) S 2D: what is being merged.

We have now added that the merge is with DAPI (now Figure S2H) in the figure and legend.

6) Fig 2G/H: statistical comparison of invasion of wt/KO should be provided.

We understand the reviewer’s point, however, our pathologist only scored the presence of invasion at the individual mouse level, which is plotted as a percentage of animals. This makes statistical analysis challenging. We would have to repeat the entire experiment two more times to get statistics (we have done pilot studies, however, we did not perform histological analysis). We also only highlight this as a trend in the text. The data are supported by the *in vitro* invasion assay in Figures 3E and 3F.

7) Fig 3A not clear what is being shown as WT and RQ seem similar at the magnification I can achieve;

Fig 3A depicts pull downs with WT, CS and RQ PTPRK mutants of the PTPRK intracellular domain. The reviewer is correct that the WT and RQ are similar, whereas the CS mutant shows a trapping effect. We and others have previously reported this for PTP CS mutants. We have unpublished data that indicates specific conformational changes that are induced by the CS mutant. The only difference between WT and RQ is in the phosphotyrosine blot, which shows slightly elevated signal in the RQ, since it has no phosphatase activity compared to the WT. The message from this experiment is that the RQ does not trap, whereas the CS does. We rationalised that this was a better mutant to use to only study the effect of catalysis. We have added extra detail to the main text and figure legend to clarify this (Page 7).

© 2024. Published by The Company of Biologists under the terms of the Creative Commons Attribution License (https://creativecommons.org/licenses/by/4.0/).

S F 3B I can not see any data for CS

We have increased the size of the graph. The data points for the CS mutant (black squares) are perfectly overlaid by the RQ (green triangles) since both show no activity in the assay. We hope the reviewer is better able to see the data points.

8) Statement referred to Fig 3C (meant S 3C) that RQ expressed to lower amount than WT needs support by statistical evaluation of multiple repeated experiments. Fig 3C. This identification of effects of PTPRK that do not involve phosphatase activity is the most innovative aspect of the study and needs more in depth study that can be statistically evaluated to support what is shown in Fig 3E,F.

Apologies for the confusion. We have sought to clarify this issue. Figure 3C shows PTPRK expression from xenograft RNA Seq samples plotted against tumour growth rate. We have now included an additional figure (S3F) comparing the expression levels of PTPRK in the xenografts. We confirm by t test that there is a difference between the induced expression of PTPRK and PTPRK.RQ at the RNA level. This could be due to differences in copy number introduced during lentiviral transduction, which is difficult to control for. This is also visible by eye in the Figure 3C western blot, where both tGFP and PTPRK levels are lower in the RQ samples from 2D cell culture.

9) Fig 3D and E as labeled represent 3E and F. Also in text, these studies are mislabeled as Fig 2 D, E

This was an error due to changing figure formats. Figure 3 labels have been corrected. In addition, the call outs in the text have been updated.

10) Fig S5, there are p values shown but in addition the legend uses * to indicate different p values.

Now corrected.

11) Fig S6B no data stated in text on ARHGAP5 are shown.

We have removed reference to ARHGAP5 since it refers to published data (page 9).

**Reviewer 2.**

Fig 3. The interpretation of the blot in Figure 3A is not entirely clear. In the text the authors indicate that these data demonstrate R1095Q mutant as phosphatase dead but not substrate trapping. Please explain why are FAM83B and DLG5 included and why do they associate with R1095Q. I suppose paxillin is included as negative control ? whereas the others are established substrates.
Please clarify this in the text.

We have now further clarified the importance of this experiment in the text (pages 6-7). This is also addressed in point 7 for reviewer 1.

Did reconstitution with R1095Q influence HT29 cell proliferation in vitro?
We did not see any differences in 10% serum using an MTS assay, which will also monitor metabolic changes. We have now included an Incucyte confluence assay (Figure 6C and D). Again, this measures both proliferation and cell spreading, but was useful for comparing with the additional scratchwound experiment (below). This showed that potentially in 10% serum, the R1095Q is not able to rescue the slightly enhanced rate to reach confluence, and actually reached confluence first out of all conditions. In contrast, with EGF, it acted in the same way as PTPRK suppressing the increased responsiveness of the PTPRK KO to EGF. The catalytic-independence is therefore relevant in the invasion assay (Matrigel contains growth factors including EGF) (Fig. 3E and F) and wherever EGF is used explicitly (see also below).

Fig 3D. Would it be possible to show the effect of R1095Q expression on junctional p-

© 2024. Published by The Company of Biologists under the terms of the Creative Commons Attribution License (https://creativecommons.org/licenses/by/4.0/).

p120 intensity similarly to the wt?

We have now included this panel. Similar to the Western blot in Figure 3D, we see an intermediate phenotype. This likely reflects the inability to directly dephosphorylate P120 catenin, however, there are also likely non-catalytic mechanisms by which PTPRK stabilises cell junctions, promoting the accumulation or expression of other RPTPs that can dephosphorylate p120, including PTPRM and PTPRJ.

Figure4. The authors cite many times Wang et al., 2005 showing that PTPRK suppresses TGFB1. However, looking at the RNAseq data there does not seem to be a significant effect in TGFB1 expression between ctrl and KO cells and TGFB is suppressed only in the reconstituted cell lines overexpressing wt or RQ PTPRK. Would be good if the authors could please comment on these data linked to the published data they discuss in 4D.

Wang et al identify PTPRK as a SMAD target gene. Our RNA seq data suggests that reciprocally, PTPRK suppresses the *TGFB1* potentially as part of a negative feedback loop. Amongst the significantly differentially regulated genes in our RNASeq dataset (Fig 4A) we identified several other TGF beta pathway associated genes, including *TACSTD2*(https://doi.org/10.1038/jid.2011.164) and *ANO1* (Anoctamin-1 is induced by TGF- beta and contributes to lung myofibroblast differentiation - PubMed (nih.gov)). We then sought to validate the findings of Wang et al in intestinal models by mining existing datasets where we can see that TGF beta does modestly stimulate PTPRK expression in organoids. This is now clarified further in the text (Page 9).

The data indicating that EGFR phosphorylation is not regulated by PTPRK are interesting and seem convincing and are in line with the increased EGFR-P upon acute stimulation S6E. As the story started with collective migration and invasion, it would be nice if the authors could test PTPRKwt vs. RQ cells for their responses to EGF-induced cell migration and/or invasion.

We agree that these data are interesting, and really go against the current notion of how this phosphatase regulates EGF signalling. We have now repeated the scratch wound assay including the rescue constructs (including Doxycycline), with and without EGF (Fig. 6A, B).This was a very interesting experiment. We repeated our previous finding, that PTPRK KO cells have impaired "healing". The results are not as striking, which might be due to the presence of doxycycline that can promote junction formation. Nevertheless, the addition of EGF completely changed the outcome. Now the PTPRK KO "heals" best, and once again we see that PTPRK or PTPRK-RQ can rescue this. Thank you for suggesting this. It really shows that there are cell behavioural consequences to the dysregulation of EGFR phosphorylation by PTPRK deletion.

We opted against repeating the invasion assay since Matrigel is likely to contain EGF already and shows catalysis independent activity of PTPRK (Figure 3E, F).

Minor 1F. Perhaps consider rewording "correctly polarized g-tubulin" with for example … check methods. IF figure legends mentions staining of GM130 but this seems to be missing from the figure ?? or are the pictures labelled PTPRK the GM130 staining?

We have corrected this. We had previously used GM130 (a Golgi marker) as a polarity marker but opted to include the gamma tubulin data. The description has now been moved to the methods as suggested.

---

Second decision letter

MS ID#: JOCES/2023/261914

MS TITLE: The receptor protein tyrosine phosphatase PTPRK promotes intestinal repair and catalysis-independent tumour suppression

© 2024. Published by The Company of Biologists under the terms of the Creative Commons Attribution License (https://creativecommons.org/licenses/by/4.0/).

AUTHORS: Katherine A Young, Katarzyna Wojdyla, Tiffany Lai, Katie E Mulholland, Silvia Aldaz Casanova, Robin Antrobus, Simon R Andrews, Laura Biggins, Betania Mahler-Araujo, Philippa Barton, Keith Anderson, Gareth W Fearnley, and Hayley J Sharpe

I am happy to tell you that your manuscript has been accepted for publication in Journal of Cell Science, pending standard ethics checks.

Reviewer 1

*Advance summary and potential significance to field*

the major advance is identifying phosphorylation independent effects of PTPRK via affecting EGF signaling. Lots of data to make this point that is not as important.

*Comments for the author*

My concerns have been addressed.

Reviewer 2

*Advance summary and potential significance to field*

The authors have addressed all of my remaining comments and their new data on the migration are very interesting. Happy to see this was a useful experiment.

*Comments for the author*

N/A

© 2024. Published by The Company of Biologists under the terms of the Creative Commons Attribution License (https://creativecommons.org/licenses/by/4.0/).
